# Therapeutic potential of compounds targeting SARS-CoV-2 helicase

**DOI:** 10.3389/fchem.2022.1062352

**Published:** 2022-12-06

**Authors:** Matthew T. J. Halma, Mark J. A. Wever, Sanne Abeln, Didier Roche, Gijs J. L. Wuite

**Affiliations:** ^1^ Department of Physics and Astronomy, Vrije Universiteit Amsterdam, Amsterdam, Netherlands; ^2^ LUMICKS B. V., Amsterdam, Netherlands; ^3^ DCM, University of Grenoble Alpes, Grenoble, France; ^4^ Edelris, Lyon, France; ^5^ Department of Computer Science, Vrije Universiteit Amsterdam, Amsterdam, Netherlands

**Keywords:** SARS-CoV-2, helicase, nsp13, drug repurposing, small-molecule inhibitors, natural products, COVID-19

## Abstract

The economical and societal impact of COVID-19 has made the development of vaccines and drugs to combat SARS-CoV-2 infection a priority. While the SARS-CoV-2 spike protein has been widely explored as a drug target, the SARS-CoV-2 helicase (nsp13) does not have any approved medication. The helicase shares 99.8% similarity with its SARS-CoV-1 homolog and was shown to be essential for viral replication. This review summarizes and builds on existing research on inhibitors of SARS-CoV-1 and SARS-CoV-2 helicases. Our analysis on the toxicity and specificity of these compounds, set the road going forward for the repurposing of existing drugs and the development of new SARS-CoV-2 helicase inhibitors.

## 1 Introduction

The global coronavirus disease (COVID-19) pandemic is caused by severe acute respiratory syndrome coronavirus 2 (SARS-CoV-2). Coronaviruses, named after the similarity of the viral capsid on microscopy to the solar corona (Author anonymous, 1968), are widespread and can cause mild infection similar to the common cold. In fact, all four human coronaviruses: HCoV-OC43, HCoV-HKU-1, HCoV-299E, and HCoV-NL63, are endemic and continuously circulate the human population (Corman et al., 2018). Three previous coronavirus outbreaks, albeit much smaller than the COVID-19 outbreak, have been reported: SARS-CoV-1, MERS-CoV, and coronavirus HuPn-2018. Similar to COVID-19, all of these are zoonotic diseases, initially transmitted to humans *via* animal hosts (Ye et al., 2020). In contrast to previous outbreaks, COVID-19 has caused massive disruptions to the lives of virtually every person since the emergence in late 2019. As of 4 November 2022, COVID-19 has caused 6.60 million deaths globally (Ritchie et al., 2020). The significant death toll and the impact on society have resulted in large-scale campaigns to develop vaccines and antivirals to prevent and combat COVID-19.

There should be no doubt about the positive outcomes of this research effort; multiple vaccines, e.g., AstraZeneca, Moderna, Pfizer/BioNTech, have been developed and deployed in many countries. The three vaccines mentioned all target the SARS-CoV-2 spike protein, either as an mRNA or inactivated adenovirus vaccine (Dai and Gao, 2021). Concerns have been raised about the emergence of vaccine-resistant SARS-CoV-2 variants, most notably the BA.4 and BA.5 omicron subvariants (Jian et al., 2022). These strains have mutations in the spike protein, and various sources report higher attack rates and infectivity for these mutants. Vaccine-produced antibodies were shown to have less neutralizing potential against omicron as compared to alpha- and delta variants (Andrews et al., 2022). Furthermore, vaccines may be less effective or even dangerous for immunocompromised individuals (Marra et al., 2022). Moreover, certain individuals are allergic to components of vaccines (Cabanillas and Novak, 2021), and adverse events are being reported (Karlstad et al., 2022). Lastly, with the likelihood of the virus to become, and remain, endemic (Lavine et al., 2021), and given the range of confirmed animal reservoirs of SARS-CoV-2 infection (Prince et al., 2021), a variety of strategies to combat SARS-CoV-2 infection are required.

### 1.1 Current antivirals

In the early days of the pandemic, there were no approved antiviral compounds against SARS-CoV-2 (World Health Organization, 2020). This changed in October 2020, when remdesivir (brand name: Veklury; Gilead Sciences) was granted emergency use authorization (EUA) by the US Food and Drug Administration (FDA) for treatment of hospitalized patients (World Health Organization 2020). Remdesivir was the only approved medicine until the EUA of molnupiravir (Merck and Ridgeback) and paxlovid (Pfizer) in December 2021 (U.S. Food and Drug Administration, 2021).

The approved drugs have different mechanisms of action; remdesivir, a nucleotide analogue, acts by stalling SARS-CoV-2 RNA-dependent RNA polymerase (RdRp) (Kokic et al., 2021). Remdesivir exhibited conflicting impact in studies, showing improvement in time to recovery in the initial study cited during authorization (Beigel et al., 2020), but later studies showed either no statistically significant effect (Wang et al., 2020), or a statistically significant but clinically minor effect (Spinner et al., 2020). Concerns over renal toxicity (Gérard et al., 2021; Wu et al., 2022), as well as a cardiac safety signal (Rafaniello et al., 2021) challenge the safety of the drug. The second drug under EUA, molnupiravir, was approved based on a study showing a reduction in hospitalization and death (Jayk Bernal et al., 2022). Molnupiravir, in addition to remdesivir, targets RNA-dependent RNA polymerase and increases the frequency of mutations during SARS-CoV-2 replication (Kabinger et al., 2021). Concerningly, it has also been shown to induce mutations in mammalian cells (Zhou et al., 2021). The mechanism of action of molnupiravir is concerning as it has a possibility of driving new variants (Kabinger et al., 2021; Hashemian et al., 2022), as a result, its use is cautioned by the World Health Organization (World Health Organization, 2022). The third approved antiviral, paxlovid acts as a 3CL protease inhibitor. 3CL protease is necessary for viral replication (Marzi et al., 2022). Paxlovid displays a reasonable safety profile, although patients often report a “paxlovid rebound” where there is a resurgence of symptoms, often worse than the initial bout (Charness et al., 2022). Moreover, drug-drug interactions have been shown to cause adverse events (Burki, 2022). Drug resistance is also a concern, as mutations have been characterized which drastically reduce the effectiveness of paxlovid (Zhou et al., 2022).

Depending on the drug target, medication is tailored for different stages in infection. Different proteins can be targeted for therapy depending on the stage of infection. Compounds targeting the spike protein will inhibit entry of SARS-CoV-2 into cells, whereas compounds targeting RNA-dependent RNA polymerase will inhibit the replication process, but will not prevent entry into the cell. Therefore, depending on the clinical course, certain compounds can be used at different stages of infection. The helicase, being a replication protein, is active in unwinding the RNA secondary structure so that it can be either replicated by RNA-dependent RNA polymerase or translated by the host ribosome.

### 1.2 Drug repurposing

Responding to emerging and pandemic viral illnesses requires a multifaceted approach, one strategy is drug repurposing. Drug repurposing is the use of approved drugs for novel targets and diseases. First, finding a useful medication amongst already existing drugs obviates the need to create novel drugs, thus saving time in disease response. Moreover, the side-effects of marketed drugs, having undergone clinical trials and prescribed use, are extensively researched and documented. Lastly, the manufacturing process is already known, and needs only to be scaled. Drug repurposing has previously found success, for example in sildenafil, an angina medication, that was successfully repurposed for erectile dysfunction as Viagra® (Pushpakom et al., 2019).

One example of a successfully repurposed and widely available medication for treatment of COVID-19 is fluvoxamine, a well-tolerated and selective serotonin reuptake inhibitor. Fluvoxamine is commonly used as an antidepressant (Sukhatme et al., 2021). It has been shown to reduce hospitalization in a large-scale randomized control trial (Reis et al., 2022). Being a repurposed drug, fluvoxamine, which was first approved by the FDA in 1994 (trade name: Luvox), has the advantage of decades of safety data surrounding its use. Unlike molnupiravir and paxlovid where a treatment course costs approximately 700 and 500 USD, respectively (Goswami et al., 2022; Morrison Ponce et al., 2022), fluvoxamine is accessible at 4 USD per course (Wang et al., 2021). Remdesivir is also expensive at over 2000 USD per 5-day treatment course (Carta and Conversano, 2021). The price and availability of drugs is an important consideration, especially considering that developing nations have far lower vaccination rates than developed nations (Bollyky et al., 2020). As of 25 July 2022, 73.2% of EU citizens have completed a full course with an EU-approved vaccine^1^ and 55.0% have received at least one booster shot (Ritchie et al., 2020). For comparison, in Africa 42.7% of individuals have been vaccinated and only 2.5% have received at least one booster shot (Ritchie et al., 2020).

Finally, other concerns shape the adoption of a particular pharmacological compound in response to a global pandemic; these include intellectual property concerns, current and future availability, distribution, and (un)known side-effects. Ultimately, an effective treatment of COVID-19 is preferred, that is widely available, inexpensive and without significant toxicity.

### 1.3 SARS-CoV-2 helicase (nsp13)

**FIGURE 1:**
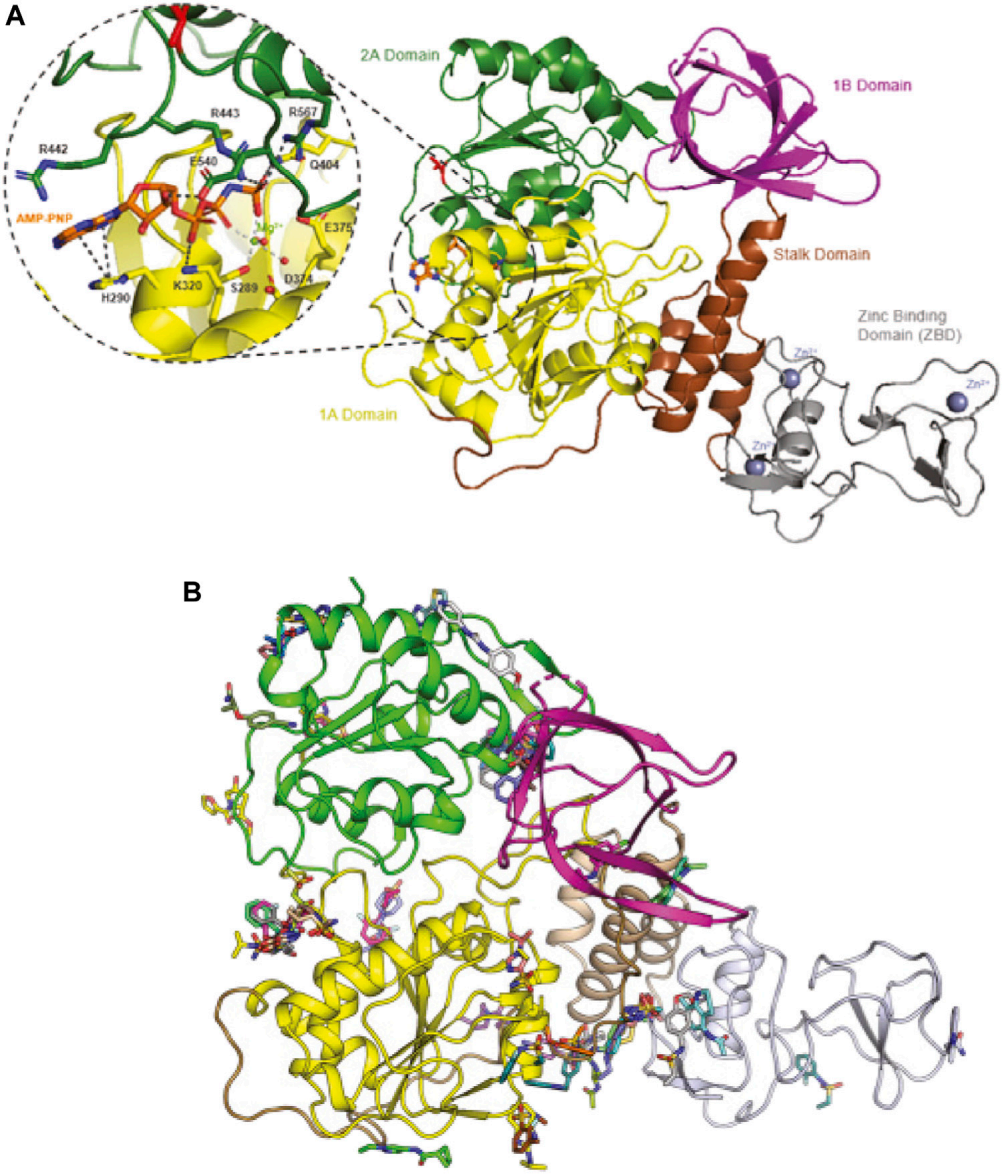
Binding sites of SARS-CoV-Nsp13 helicase. Panel **(A)** Structure of SARS-CoV-Nsp13 helicase (PDB ID: 7NN0) (Newman et al., 2021). V570, the single different residue from SARS Helicase (I570) is highlighted in red. The residues constituting the ATP binding site are shown in the enlarged window bound with AMP-PNP, an AMP analog. Panel **(B)** Possible binding pockets from Nsp13 fragment screening. Reproduced from Newman et al., 2021 under a Creative Commons Attribution 4.0 International License (http://creativecommons.org/licenses/by/4.0/).

Drug repurposing is mostly a phenotypic approach, meaning that protein target and mechanism of action are often unknown. In contrast, target-based approaches seek to first identify protein targets (chemical biology) and to subsequently develop small-molecule inhibitors (medicinal chemistry) for the target. In principle, every SARS-CoV-2 protein can be considered a target, but it is preferable to target essential and/or conserved proteins. A previous review has already reviewed and postulated the main drug targets for COVID-19 (Gil et al., 2020), while this report focuses on the helicase of SARS-CoV-2. The SARS-CoV-2 nsp13 gene encodes a molecular motor, which is a 5′ to 3′-translocating helicase, belonging to superfamily 1B. Helicases act on (deoxy)-ribonucleic acid substrates and are fueled by (deoxy)-nucleotide triphosphates (Figure 1A). The primary functions of helicases are in DNA repair, replication, recombination, and transcription.

Nsp13 is one of the most conserved genes in the SARS-CoV-2 genome, having one of the lowest mutation rates of any of the essential SARS-CoV-2 proteins (Martin et al., 2021; Newman et al., 2021). The SARS-CoV-2 helicase differs from the SARS-CoV-1 helicase by only one amino acid residue, i.e., V570 in SARS-CoV-2 helicase (Figure 1A, highlighted in red) compared to I570 in SARS-CoV-1 helicase, allowing drugs discovered for SARS-CoV-1 to potentially be re-used. Potential binding pockets of Nsp13 were explored *via* crystallographic fragment screening (Figure 1B), presenting a starting point for structure-based drug discovery (Newman et al., 2021). Moreover, the helicase plays a critical role in replication of the viral genome (Jia et al., 2019). The combination of these two argues for the functional importance of SARS-CoV-2 helicase and makes it an attractive target for the development of antivirals. This is also evidenced by an upcoming CACHE challenge^2^ that aims to discover new molecules that target SARS-CoV-2 helicase.

The viral helicase is not a new target in drug discovery, for example the helicases of herpes simplex virus and hepatitis C virus have been targeted, as reviewed by Shadrick et al. (Shadrick et al., 2013). More recent reports feature the helicases of polyomaviruses, Zika virus, and MERS-CoV (Bonafoux et al., 2016; Kumar et al., 2020; Zaher et al., 2020; Mehyar et al., 2021b). Additionally, human helicases have also attracted research interest, and inhibitors for DDX and BLM, among others, have been reported (Datta and Brosh, 2018). This approach aims to use small molecule inhibitors to sensitize cancer cells to chemotherapy and DNA-damaging agents and/or to utilize specific tumor backgrounds for hypersensitization of tumors to pharmacological inhibition, a concept which is known as synthetic lethality (Datta and Brosh, 2018).

## 2 Main considerations

### 2.1 Target stability

**FIGURE 2:**
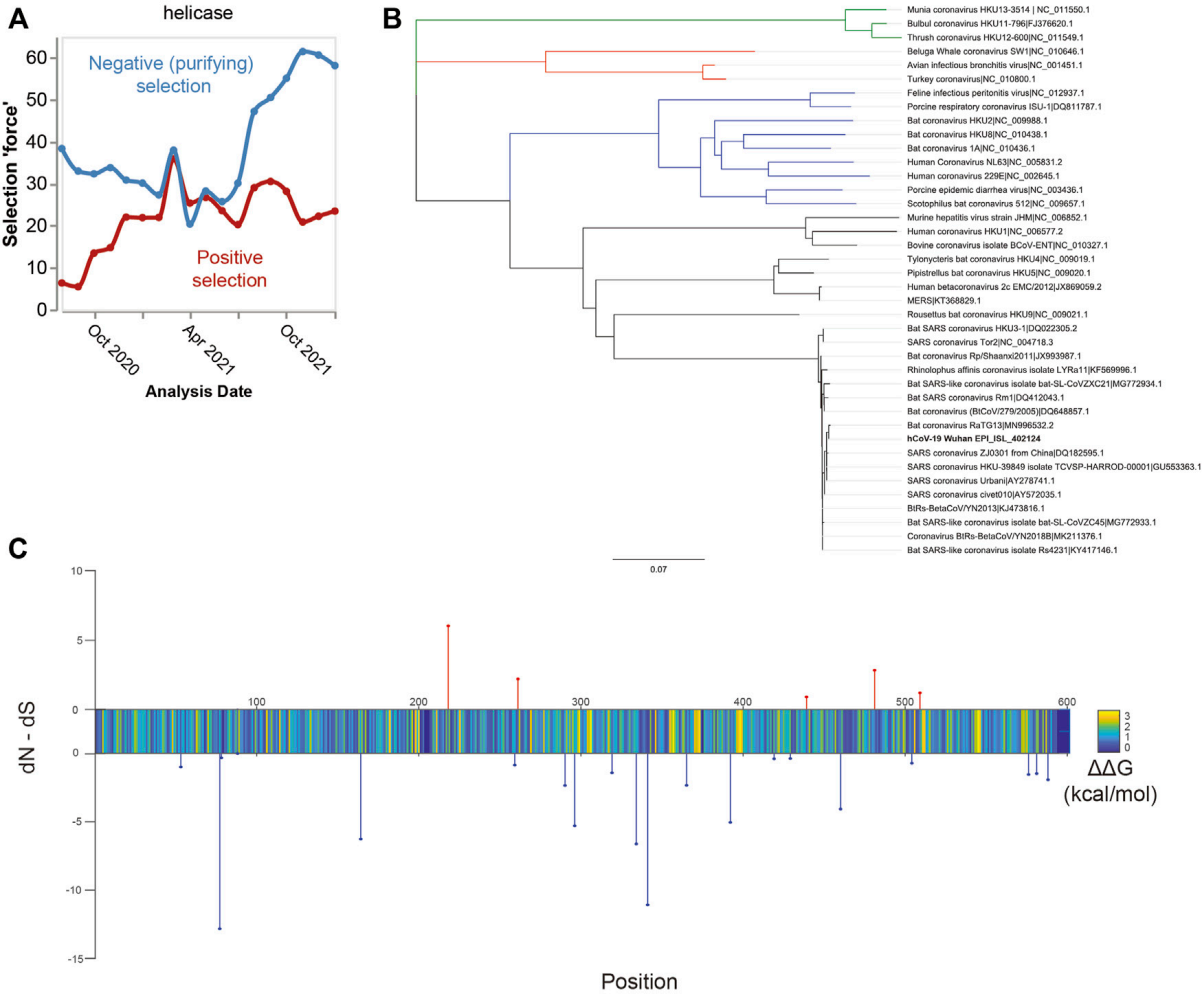
Suitability of the nsp13 protein as a drug target. Panel **(A)** Time course of selection pressures on SARS-CoV-2 helicase from August 2020 to January 2022. Blue lines show the extent of negative selection, defined as the number of sites under negative selection normalized by kilobase of gene length and the internal tree length. Red lines how the positive selection force, defined as the number of positively selected sites with the same normalization. Over the time history, more sites show negative (purifying) selection, suggesting evolutionary stability. Panel **(B)** Phylogenetic tree of the coronavirus family based on nsp13 protein sequences. Legend: alpha-CoV (blue), beta-CoV (black), delta-CoV (red), and gamma-CoV (green). Within the beta-CoVs, there is high nsp13 conservation shown by the short tree lengths. Given the low variance amongst this clade, it may be possible that a SARS-CoV-2 nsp13 inhibitor also inhibits the other clade members. Panel **(C)** Energetics and selection on residues in SARS-CoV-2 nsp13 helicase. Stem plots show positive (red) or negative (blue) selection, expressed as FEL rate. Color plot shows the average energetic change in kcal/mol of all mutations at the site.

As previously mentioned, SARS-CoV-2 helicase is among the most conserved proteins in the SARS-CoV-2 genome (Martin et al., 2021; Newman et al., 2021). Throughout the pandemic, it has remained largely stable. Phylogenetic evidence demonstrates increasing negative, i.e., purifying, selection over time, making it a stable target (Figure 2A). The development of drug resistance is an issue that undermines many treatments, most notably anti-biotics. Under the selection pressure of a drug treatment, the target protein can mutate such that the compound no longer binds (Richman, 1994; Menéndez-Arias and Richman, 2014). It was evaluated whether the mutations observed through genomic surveillance of COVID-19 cases (Kumari et al., 2022) altered the initial protein sequence (Newman et al., 2021). For a drug to retain effectiveness over time, the major mutations would not alter binding affinity of the drug compounds, thus maintaining drug effectiveness against mutations. Possibly, conservation of structure may enable production of pan-beta coronaviral inhibitors to guard against future zoonotic coronaviral outbreaks (Li et al., 2021; Munshi et al., 2022). This possibility is supported by the low level of nsp13 genetic variation within beta-coronaviruses, as demonstrated by the phylogenetic tree shown in Figure 2B.

The technique used here to identify mutations is exploratory, in that the predicted energetic shift was used as a proxy for conformational change. It has been assessed whether there are any changes likely to significantly impact the structural conformation of SARS-CoV-2 helicase. If a mutation was near a binding site and significantly shifted the energetic stability of the protein, it is likely that the mutation alters compound binding. Selection was determined using the toolkit made from the GISAID database^3^, for all SARS-CoV-2 genomes up to 2 January 2022. In Figure 2C, site selection in terms of fixed-effects likelihood (FEL) (Kosakovsky Pond and Frost, 2005) is displayed (blue and red stem plots), FEL is a measure of selection pressure in phylogenetic trees and is calculated by comparing the expected number of non-synonymous mutations with the actual observed rate. In short, observing a higher than expected frequency of non-synonymous mutations suggests positive selection, *i.e.*, evolutionary pressure for the protein to change. Observing fewer than expected non-synonymous mutations is evidence of negative purifying selection, whereby mutants are not likely to survive and reproduce.

Additionally, a color plot depicts the average change in energetic stability of the protein resulting from the set of possible mutations at that site (Kwasigroch et al., 2002). Most mutations result in a slight destabilization of the helicase protein, suggesting a high level of structural optimization. While this makes it less likely that the protein will develop a drug resistant mutation, it is not certain. Residues where mutations have a destabilizing effect are more likely to alter the helicase structure, which affects the binding of compounds. The limitation of this approach is the lack of experimental data to support the generated model. Our assessment shows potentially worrisome loci for future drug resistance, where there is a confluence of positive selection and an energetically destabilizing impact (upward stems in Figure 2C). These sites should be monitored for development of drug resistance and ideally a drug will either act on a different location, or the destabilization is significant enough to render the protein non-functional.

### 2.2 Current inhibitors: *In vitro*, *in vivo* and *in silico* assessment

Having established the validity of the helicase as a drug target, multiple methods can be applied for the discovery of inhibitors. *In silico* screening is experimentally less intense, requiring mostly computational power. This methodology requires the availability of an X-ray or cryo-EM structures. The crystal structure for SARS-CoV-1 helicase was solved in 2019 (Jia et al., 2019), whereas for SARS-CoV-2 helicase structural information was first published in 2021 (Newman et al., 2021). Earlier *in silico* research made use of homology models based on either SARS-CoV-1 or MERS-CoV helicase to perform molecular modelling studies. Orthogonal to *in silico*, is *in vitro*, the screening of compounds directly on the protein of interest. This methodology can be low- (1–100), medium- (100–10.000) or high- (>10.000) throughput, depending on the equipment used and assay deployed. The most common *in vitro* assay performed for helicases is an ATP-turnover assay, there is, however, a high risk for false positives, e.g., aggregators or DNA-binders, when running these experiments (McGovern et al., 2003; Acker and Auld, 2014). Another common *in vitro* assay for helicase activity is to measure the unwound fraction by using a DNA construct with a double stranded region formed by an annealed oligonucleotide. If the helicase is active, it will separate the oligonucleotide from the construct, and a lighter band will show up on the gel. Form the intensity of this band, the unwound fraction and subsequent helicase activity can be calculated (Kim and Seo, 2009).

#### 2.2.1 SARS-CoV-1 helicase

The first reports of compounds with SARS-CoV-1 helicase activity date back to 2005, when Tanner et al., described a group of adamantane-derived bananins (**1**-**4**, Supplementary Table S1, Supplementary Figure S1) with low micromolar ATPase and helicase inhibitor activities (Tanner et al., 2005). These pyridoxal-conjugated trioxa-adamantanes were shown to be non-competitive inhibitors by DNA- and ATP-competition assays and did not exhibit inhibitory activity on *E. coli* DnaB helicase. To the best of our knowledge, compounds **1**-**4** have not been further investigated. Structurally different Ranitidine Bismuth Citrate (**5**, Supplementary Table S1) inhibits ATPase and DNA-duplex unwinding activity, IC_50_ = 0.3 and 0.6 µM, respectively (Yang et al., 2007b). Compound **5** is the most potent from a series of bismuth complexes (Yang et al., 2007a), whose mechanism of action involves the displacement of Zinc ions from the ATP-binding site (Yuan et al., 2020). Furthermore, flavonoids have been shown to inhibit SARS-CoV-1 helicase. Myricetin (**6**), baicalein (**7**), quercetin (**8**), and scutellarein (**9**) all are natural products that inhibit helicase and/or ATPase activity in the low micromolar range (Lee et al., 2009b; Yu et al., 2012; Keum et al., 2013). Flavonoids have been ascribed many potential health benefits, including antineoplastic and antiviral. However, there have also been multiple reports characterizing flavonoids as false positives and protein aggregators in biological assays. Myricetin (**6**) has been reported to inhibit many other targets including *E. coli* DnaB helicase and DNA polymerase (Griep et al., 2007). The activity of flavonoids on SARS-CoV-1 helicase has further been validated by the design and synthesis of compounds **10**–**15** (Lee et al., 2009b; Kim et al., 2011). There is still a requirement for further experimentation to investigate the inhibition and selectivity of flavonoids and synthetic analogues thereof on SARS-CoV-1 helicase. Aryl di-keto acids are derived from flavonoids, and were also shown to inhibit SARS-CoV-1 helicase and various other targets, *e.g.,* hepatitis C virus RNA polymerase (Lee et al., 2009a). Lastly, four compounds (**17**–**20**) have been published but there was no information on related compounds. SSYA-10–001 (**18**) has additionally been reported as an inhibitor of hepatitis C virus RNA polymerase and MERS-CoV helicase (Adedeji et al., 2012, 2014).

#### 2.2.2 SARS-CoV-2 helicase

The first reports on inhibitors of SARS-CoV-2 helicase were compounds that have previously been investigated for SARS-CoV-1 helicase, namely bismuth complexes (**5**, **21**–**24**) (Supplementary Table S2, Supplementary Figure S2). Ranitidine Bismuth Citrate (**5**) was validated with sub-micromolar helicase and ATPase IC_50_’s (Yuan et al., 2020) and exhibited greater activity compared to Bismuth (III) tetraphenylpoprhyrinate (**23**) and Bismuth (III) tetra-4-pyridiylporphyrinate (**24**). Moreover, **5** relieved virus-associated pneumonia in a golden Syrian hamster model. Disulfiram (**25**) and Ebselen (**26**) (Supplementary Table S2) are other Zinc-ejector drugs that have been validated on SARS-CoV-2 helicase (Chen et al., 2021).

White et al. have identified a hit list of 368 FDA-approved drugs, from which cepharanthine (**27**), IC_50_ = 400 µM and lumacaftor (**28**), IC_50_ = 300 µM) were confirmed in an ATPase assay (White et al., 2020). Cepharanthine (**27**) has previously been reported as a SARS-CoV-1 inhibitor, however at the time the target enzyme was not known (Zhang et al., 2005). Vapreotide (**29**), grazoprevir (**30**) and simeprevir (**31**) are other FDA-approved drugs discovered by phenotypic screening that inhibit SARS-CoV-2 helicase *in vitro*. Their activities were confirmed by a DNA-unwinding activity assay with IC_50_ values of ≈10, ≈2.5, and ≈1.25 µM, respectively (Muturi et al., 2022). All three compounds have also been reported as virtual hits (Borgio et al., 2020; Gurung, 2020). Furthermore, a high-throughput screening of five thousand known pharmaceuticals by Zeng et al., mentions the inhibitory activity of FPA124 (**32**), IC_50_ = 8.5 µM) and suramin (**33**, IC_50_ = 0.94 µM). These hits were confirmed by a fluorescence resonance energy transfer (FRET) based helicase assay in the presence of Tween-20. Tween-20 is a non-ionic detergent that stops potential colloid formation. Both compounds still inhibited helicase activity in this assay at IC_50_ = 8.4 µM and 1.1 µM, respectively, and viral inhibition was confirmed *in vivo* on Vero E6 cells (Zeng et al., 2021). SARS-CoV-1 inhibitors myricetin (**6**) and SSYA-100–01 (**18**) were used as a comparison in these experiments and were confirmed to be active on SARS-CoV-2 helicase. Research from the EXSCALATE4COV (E4C)^4^ project on a natural product library once more confirmed the activity of SSYA-100–01 (**18**) and identified five flavonoids with low micromolar activity: myricetin (**6**), quercetin (**8**), kaempferol (**34**), flavanone (**35**), and licoflavone C (**36**) (Corona et al., 2022). Moreover, Mehyar et al. (2021a) report on the repurposing of sulphoxide- and sulphone-containing FDA-approved compounds. Zafirlukast (**37**) was the only compound with inhibitory activity, interestingly **37** was also reported by Zeng et al., 2021), but was not selected for further analysis (Zeng et al., 2021). Mehyar et al. (2021a) also report SARS-CoV-2 helicase inhibitory activity for five previously identified MERS-CoV helicase inhibitors (**37**–**42**). Lastly, Newman et al. identified 65 fragments by crystallographic fragment screening. Although there were no inhibitory values published for these fragments, the crystal structures show binding in the ATP binding site as well as the RNA/DNA-entry tunnel. These crystal structures have been made publicly available and can be seen as a starting point for fragment growing (Newman et al., 2021). More recently, Romeo *et al.* identified multiple inhibitors with predicted binding to the RNA/DNA-entry tunnel *in vitro*. (Romeo et al., 2022).

Although *in vitro* and *in vivo* assays are the gold standard for hit validation, virtual screening allows for rapid identification of ‘virtual’ hits. The screening of ultra-large chemical spaces *in silico* has greatly increased the possibilities of modern drug discovery (Warr et al., 2022), but biological assays are still required to validate these hits. Not all laboratories, however, have the means to perform *in vitro* assays, thus making molecular modeling a more accessible method for initial target investigation. The SARS-CoV-2 helicase has been screened, virtually, in many instances (Supplementary Table S3). From our analysis it was observed that most publications have performed virtual screening on commercially available drugs (Balasubramaniam and Schmookler Reis 2020; Borgio et al., 2020; Gurung 2020; Iftikhar et al., 2020; Ugurel et al., 2020; Abidi et al., 2021; Sundar et al., 2021; Alanazi et al., 2022; Azmoodeh et al., 2022) or natural products (Kousar et al., 2020; Naik et al., 2020; Ahmad et al., 2021; James et al., 2021; Vivek-Ananth et al., 2021; Bhargavi et al., 2022; Hossain et al., 2022; Samdani et al., 2022). Other published works make use of fragments (Freidel and Armen, 2021) or publicly available compound libraries (Mirza and Froeyen, 2020; García et al., 2021; El Hassab et al., 2022; Pitsillou et al., 2022). It is recognized that multi-targeted approaches are often carried out, most notably including RNA-dependent RNA polymerase and 3CL protease, to have dual-target SARS-CoV-2 inhibitors. The best scoring helicase inhibitors resulting from *in silico* approach, and without *in vitro* data, are shown in Supplementary Table S3. One particularly large study performed ultra-large virtual screening of one billion molecules on fifteen SARS-CoV-2 proteins, for each target the top 1,000 and top one million (0.1%) are publicly available online^5^ (Gorgulla et al., 2021). All publications mentioned in this paragraph, however, lack the biological validation that is required to confirm activity. The occurrence of false positives in virtual screening is still high and results do often not translate to *in vitro* assays, as was recently shown by Cerón-Carrasco (Cerón-Carrasco, 2022). Thus, it remains critical to validate ‘virtual’ hits and to refrain from the use of thereof in determining structure-activity relationships.

### 2.3 Toxicity analysis

The potential side-effects of any treatment are a concern for medical practitioners when making a choice of which therapy to implement. Certainly, drugs with minimal off-target toxicity are preferred. While toxicity information exists for some compounds in the included tables, many have limited application as treatments and therefore little associated data on side-effects. Toxicity prediction applies machine learning to chemical structures with known toxicity tests on model organisms. Based on chemical similarities, the toxicity of untested compounds can be predicted. Toxicity prediction is a useful tool for evaluating potential harmful side-effects before taking the drug through costly pre-clinical and clinical trials.

It was not possible to use the same assay or toxicity prediction for all compounds. Individual studies often use different assays and thus report different values. Additionally, the toxicity prediction software was not always successful, and therefore several different tools were used: the Quantitative Structure-Activity Relationship (QSAR) toolbox, developed by the Organization for Economic Cooperation and Development (OECD) (Dimitrov et al., 2016); the Toxicity Estimation Software Tool (TEST) software developed by the US Environmental Protection Agency (US-EPA) (Martin et al., 2008); and the lazar toxicity prediction web server (Maunz et al., 2013). For some compounds, particularly pharmaceutical drugs, toxicity data was accessible from public documents for their approval by either the FDA or the European Medicines Agency. Many of the natural products included have long histories of use in food as well as herbal medicines (Wang and Yang, 2020, 2021; Yang and Wang, 2021; Wang et al., 2022). Many are found in common foods and show strong association with positive health outcomes (Kumar and Pandey, 2013), including possible antiviral and antineoplastic (Rodriguez-García et al., 2019) properties. Since these products have been consumed for millennia, it is unlikely that they exhibit toxicity, although this may of course be different when the active compound becomes highly concentrated. The retrieved experimental toxicities and/or the predicted toxicity values for every compound are provided in Supplementary Tables S1,S2,S3 for the reader’s consideration. For most assays, acute toxicity values were reported, this certainly has its drawbacks, as compounds may exhibit toxicity at much lower doses. These toxicities should not be overly interpreted, since the effective IC_50_ doses of compounds differ, it is more beneficial to take a selective ratio against a toxicity endpoint.

## 3 Discussion

In Supplementary Tables S1,S2,S3, the source information of SARS-CoV-2 inhibitors is found, referring to where the compound can be extracted, synthesized or otherwise procured. Three categories are presented: Natural Products (NP), Synthetic Products (SP) and Pharmaceutical Drugs (PD). Natural products need only be extracted from their source organism, typically a plant; pharmaceutical drugs are approved molecules for the treatment of diseases, though some may be off-market. Synthetic products are typically only produced in very specific contexts, typically a research study. For natural products, the source organism(s) are indicated, whereas for pharmaceutical drugs the tradename and manufacturers are mentioned. Contrary to natural products and pharmaceutical drugs, synthetic products often do not yet have a known toxicity profile.

**FIGURE 3:**
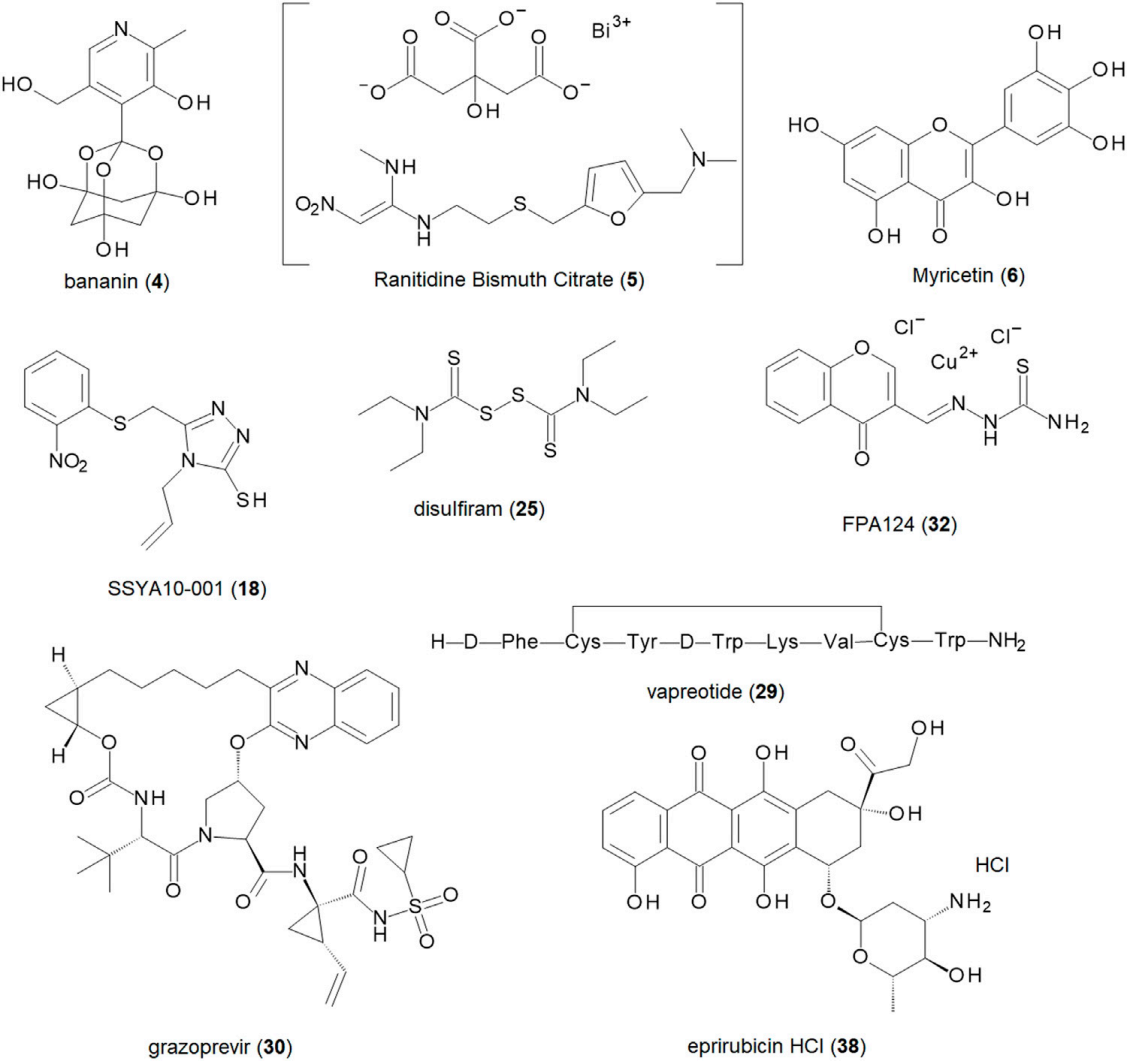
Structures of the nine most promising SARS-CoV-2 helicase inhibitors for further development and drug repurposing.

From the compounds in Supplementary Tables S1,S2, the nine most promising compounds for further development are shown in Table 1 and Figure 3. They have been determined based on inhibitory activity, number of orthogonal assays and structural diversity. The first compound, bananin, was discovered, along with several other related compounds, to inhibit the helicase of SARS-CoV-1 (Tanner et al., 2005). As such, it presents a scaffold on which lead optimization can be performed. Two other synthetic products, SSYA10-001 and FPA124, offer promising scaffolds to develop into pharmaceutical drugs, should they have a reasonable biodistribution and safety profile. Ranitidine Bismuth Citrate (RBC) is a promising compound showing inhibition in helicase unwinding assays, as well as *in vivo* activity in a Syrian hamster model (Yuan et al., 2020). RBC has a higher level of validation than the other compounds, and its previous use as a pharmaceutical (TRITEC, GlaxoSmithKline) make it a promising drug for repurposing. Other pharmaceutical drugs for potential repurposing are disulfiram, vapreotide and grazoprevir. These are distinct enough that they can be developed as independent scaffolds. Among the natural products, myricetin, has the lowest IC_50_ (0.41 µM) of flavonoid compounds against SARS-CoV-2 (Supplementary Table S2). Its safety, wide use, and availability make it a promising compound for development. Another natural product, Epirubicin HCl, is included for its low IC_50_ (0.31 µM), while still being distinct enough from myricetin to develop it as a distinct scaffold.

**TABLE 1:** the nine most promising SARS-CoV-2 helicase inhibitors for further development and drug repurposing.

Name (#)	Classification	Reference
Bananin (**4**)	synthetic product	Tanner et al. (2005)
Ranitidine Bismuth Citrate (**5**)	pharmaceutical drug	Yang et al. (2007b)
		Yuan et al. (2020)
Myricetin (**6**)	natural product	Yu et al. (2012)
		Zeng et al. (2021)
		Corona et al. (2022)
SSYA10-001 (**18**)	synthetic product	Adedeji et al. (2012)
		Zeng et al. (2021)
		Corona et al. (2022)
Disulfiram (**25**)	pharmaceutical drug	Chen et al. (2021)
Vapreotide (**29**)	pharmaceutical drug	Borgio et al. (2020)
		Muturi et al. (2022)
Grazoprevir (**30**)	pharmaceutical drug	Gurung (2020)
		Muturi et al. (2022)
FPA124 (**32**)	synthetic product	Zeng et al. (2021)
Epirubicin HCl (**38**)	natural product	Mehyar et al. (2021b)

This review summarizes and builds on the work on discovery of therapeutics targeting SARS-CoV-2 helicase, a vital replication protein. We demonstrate that this protein is highly conserved and resistant to drug-inactivating mutations. Additionally, the high degree of conservation within the coronavirus family, and particularly the beta-coronavirus clade, make coronaviral helicases attractive targets for future coronaviral outbreaks.

We have aimed to provide a complete overview of drugs, natural products, and synthetic products targeting the SARS-CoV-2 helicase, at several levels of discovery. A broad range of compounds either computationally predicted to bind to the target or with higher levels of validation, such as *in vitro* or even *in vivo* assays, have been covered. Furthermore, a summary of clinical trials for COVID-19 that involve these compounds can be found as Supplementary Table S4. Toxicity information on compounds was provided and predicted for those with absent literature values.

Overall, SARS-CoV-2 helicase is an attractive drug target for COVID-19. The potential of immune escape of future SARS-CoV-2 strains from the immunity imparted by the current vaccination program motivates the development of backup treatment options (Harvey et al., 2021; Lazarevic et al., 2021). Finally, while vaccines are a preventive measure, there is still a need for acute therapeutic interventions, for which there is currently a paucity of options. Both targeting the SARS-CoV-2 helicase by drug repurposing or new drug discovery may provide acute interventions for COVID-19 in the future.

## Data Availability

The original contributions presented in the study are included in the article/Supplementary Material, further inquiries can be directed to the corresponding author.

## Footnotes

1 i.e., Two doses of Moderna MRNA-1273, two doses of Pfizer-BioNTech BNT162B2, two doses of Oxford-AstraZeneca ChAdOx1 or a single dose of Johnson & Johnson Ad26.COV2.S.

2
https://cache-challenge.org/competitions/competition-2.

3
https://observablehq,com/@spond/revised-sars-cov-2-analytics-page.

4
www.exscalate4cov.eu.

5 See: https://vf4covid19.hms.harvard.edu/.
