# Supplementary material for: Therapeutic potential of compounds targeting SARS-CoV-2 helicase
Source: Front Chem. 2022 Dec 6;10:1062352. doi: 10.3389/fchem.2022.1062352 (PMC9763700; doi:10.3389/fchem.2022.1062352)
Supplement: Supplementary file 1 [file DataSheet1.docx]

Supplementary Material

**Supplementary table 1.** SARS-CoV-1 helicase inhibitors validated *in vitro*.

| **Name (#)^a^** | **Activity** | **Toxicity^b,c,e^** | **Classification^d^** | **Source^e^** | **Indication^e^** | **Reference** |
| --- | --- | --- | --- | --- | --- | --- |
| Vanilinbananin (**1**) | Helicase IC_50_ = 2.7 µM  ATPase IC_50_ = 0.68 µM | LD_50_ (EPA) = 1371 mg/kg | SP |  |  | (Tanner et al., 2005) |
| Iodobananin (**2**) | Helicase IC_50_ = 7.0 µM  ATPase IC_50_ = 0.54 µM | LD_50_ (EPA) = 19691 mg/kg | SP |  |  | (Tanner et al., 2005) |
| Eubananin (**3**) | Helicase IC_50_ = 5.4 µM  ATPase IC_50_ = 2.8 µM | LD_50_ (EPA) = 76 mg/kg | SP |  |  | (Tanner et al., 2005) |
| Bananin (**4**) | Helicase IC_50_ = 3.0 µM  ATPase IC_50_ = 2.3 µM | exp. CC_50_ = 390 µM  LD_50_ (EPA) = 389 mg/kg | SP |  |  | (Tanner et al., 2005) |
| Ranitidine Bismuth Citrate (**5**) | Helicase IC_50_ = 0.3 µM  ATPase IC_50_ = 0.6 µM | exp. CC_50_ = 5.0 mM  exp. CC_50_ = 2243 µM  LD_50_ (hamster, I.P.) > 150 mg/kg/day | PD | TRITEC  **GlaxoSmithKline**  (Off-market since 1999) | Ulcers due to *H. pylori* | (Yang et al., 2007b)  (Yuan et al., 2020) |
| Myricetin (**6**) | ATPase IC_50_= 2.71 µM | exp. LD_50_ (Mouse, I.P.) = 1410 mg/kg  LD_50_ (EPA) = 1636 mg/kg | NP (flavonoid) | Many natural sources, e.g., tomatoes and oranges | Many reported effects, e.g., anti-oxidative and hypo-glycemic | (Yu et al., 2012) |
| Baicalein (**7**) | ATPase IC_50_ = 0.47 µM | exp. CC_50_ = 32.9 µM  LD_50_ (EPA) = 1270 mg/kg  *Phase 1 clinical trial:* “Single oral doses of 100-2800 mg of baicalein were safe and well tolerated by healthy subjects” | NP (flavonoid) | *Scutellaria baicalensis* | Many reported effects, e.g., anti-oxidative and anti-inflammatory | (Li et al., 2012;  Li et al., 2014;  Keum et al., 2013) |
| Quercetin (**8**) | Helicase IC_50_ = 8.1 µM  ATPase IC_50_ > 50 µM | exp. LD_50_ (mouse, oral) = 160 mg/kg  LD_50_ (EPA) = 1606 mg/kg | NP (flavonoid) | Many natural sources, e.g., capers and kale | Many reported effects, e.g., anti-oxidative and antibacterial | (Lewis and Sax, 2004; Lee et al., 2009b) |
| Scutellarein (**9**) | ATPase IC_50_ = 0.86 µM | LD_50_ (EPA) = 1308 mg/kg | NP (flavonoid) | Scutellaria baicalensis |  | (Yu et al., 2012) |
| (**10**) | Helicase IC_50_ = 8.1 µM  ATPase IC_50_ = 42.9 µM | LD_50_ (EPA) = 774 mg/kg | SP |  |  | (Lee et al., 2009b) |
| (**11**) | Helicase IC_50_ = 9.3 µM  ATPase IC_50_ > 50 µM | LD_50_ (EPA) = 1281 mg/kg | SP |  |  | (Lee et al., 2009b) |
| (**12**) | Helicase IC_50_ = 2.7 µM  ATPase IC_50_ = 25.4 µM | exp. CC_50_ = 21.1 µM  LD_50_ (EPA) = 698 mg/kg | SP |  |  | (Lee et al., 2009b; Park et al., 2012) |
| (**13**) | Helicase IC_50_ = 5.2 µM  ATPase IC_50_ > 50 µM | exp. CC_50_ = 17.8 µM  LD_50_ (EPA) = 981 mg/kg | SP |  |  | (Lee et al., 2009b; Park et al., 2012) |
| (**14**) | Helicase IC_50_ = 4.1 µM  ATPase IC_50_ = 20.9 µM | exp. CC_50_ = 18.2 µM  LD_50_ (EPA) = 910 mg/kg | SP |  |  | (Lee et al., 2009b; Park et al., 2012) |
| 6-(3-chloro-benzyloxy)-5-hydroxy-2-(3-iodo- benzyloxy)-chromen-4-one (**15**) | Helicase IC_50_ = 11.0 µM  ATPase IC_50_ = 4.0 µM | exp. CC_50_ > 50 µM  LD_50_ (EPA) = 128 mg/kg | SP |  |  | (Kim et al., 2011) |
| (Z)-4-(3-((4-chlorobenzyl)oxy) phenyl)-2-hydroxy-4-oxobut-2-enoic acid (**16**) | Helicase IC_50_ = 13.6 µM  ATPase IC_50_ = 24.4 µM | LD_50_ (EPA) = 638 mg/kg | SP |  |  | (Lee et al., 2009a) |
| HE602 (**17**) | ATPase IC_50_ = 6.9 µM | exp. TC_50_ > 50 µM  LD_50_ (EPA) = 1033 mg/kg | SP |  |  | (Kao et al., 2004) |
| SSYA10-001 (**18**) | Helicase IC_50_ = 6.0 µM  ATPase IC_50_ > 100 µM | exp. CC_50_ > 250 µM  LD_50_ (EPA) = 1198 mg/kg  LD_50_ (QSAR) = 120 mg/kg/day | SP |  |  | (Adedeji et al., 2012, 2014) |
| (E)-3-(furan-2-yl)-N-(4-sulfamoyl phenyl)acrylamide (**19**) | Helicase IC_50_ = 13.2 µM  ATPase IC_50_ = 2.09 µM | exp. CC_50_ > 40 µM  LD_50_ (EPA) = 7626 mg/kg  LD_50_ (QSAR) = 520 mg/kg/day | SP |  |  | (Lee et al., 2017) |
| 7-ethyl-8-mercapto-3-methyl-3,7-dihydro-1H-purine-2,6-dione (**20**) | Helicase IC_50_ = 41.6 µM  ATPase IC_50_ = 8.66 µM | exp. CC_50_ > 80 µM  LD_50_ (EPA) = 1203 mg/kg | SP |  |  | (Cho et al., 2015) |

^a^ SMILES can be found in supplementary table 5. ^b^ Experimental (exp.) toxicity is referenced, predicted toxicity is displayed as: LD_50_ (mouse, oral) from a single injection after 48 hours predicted by US EPA TEST Software (Martin et al., 2008) or LD_50_ (mouse, oral) predicted by QSAR Toolbox (Dimitrov et al., 2016). ^c^ I.P. = intraperitoneal, I.V. = intravenous. ^d^ SP = Synthesized Product; NP = Natural product; PD = Pharmaceutical Drug. ^e^ Where possible taken from PubChem (Kim et al., 2019) and ChEBI (Hastings et al., 2016).


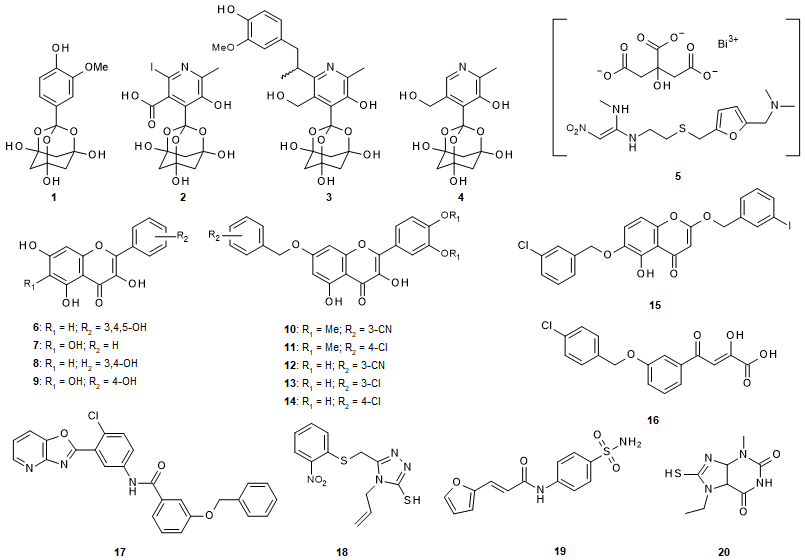


**Supplementary figure 1**: Chemical structures of SARS-CoV-1 inhibitors validated *in vitro*.

**Supplementary table 2.** SARS-CoV-2 helicase inhibitors validated *in vitro*.

| **Name (#)^a^** | **Methodology** | **Activity** | **Toxicity^b,c,e^** | **Classification^d^** | **Source^e^** | **Indication^e^** | **Reference** |
| --- | --- | --- | --- | --- | --- | --- | --- |
| Ranitidine Bismuth Citrate (**5**) | *in vitro*  *in vivo* | Helicase IC_50_ = 0.70 µM  ATPase IC_50_ = 0.69 µM EC_50_ (Vero E6) = 2.3 µM | exp. CC_50_ = 5.0 mM  exp. CC_50_ = 2243 µM  LD_50_ (hamster, I.P.) > 150 mg/kg/day | PD | TRITEC  **GlaxoSmithKline**  (Off-market since 1999) | Ulcers due to *H. pylori* | (Yang et al., 2007a; Yuan et al., 2020) |
| Bismuth Citrate (**21**) | *in vitro* | *No value provided* | LD_50_ (QSAR) = 5000 mg/kg/day | PD | PYLERA  **Aptalis**  Note: Combination of bismuth subcitrate, metronidazole, and tetracycline | Ulcers due to *H. pylori* | (Shu et al., 2020; Yuan et al., 2020) |
| Bismuth Potassium Citrate (**22**) | *in vitro* | *No value provided* | MRDD (Lazar) = 410.0 mg/kg/day | SP |  |  | (Shu et al., 2020; Yuan et al., 2020) |
| Bismuth(III) tetraphenylporphyrinate (**23**) | *in vitro  in vivo* | Helicase IC_50_ = 3.69 µM  ATPase IC_50_ = 2.39 µM EC_50_ (Vero E6) = 3.9 µM | exp. CC_50_ > 400 µM | SP |  |  | (Yuan et al., 2020) |
| Bismuth(III) tetra-4-pyridylporphyrinate (**24**) | *in vitro  in vivo* | Helicase IC_50_ = 2.64 µM  ATPase IC_50_ = 4.68 µM EC_50_ (Vero E6) = 7.5 µM | exp. CC_50_ > 400 µM | SP |  |  | (Yuan et al., 2020) |
| Disulfiram (**25**) | *in vitro* | ATPase IC_50_ = 0.41 µM | LD_50_ (EPA) = 479 mg/kg | PD | Antabuse  **Odyssey Pharmaceuticals** | alcohol use disorder | (Chen et al., 2021) |
| Ebselen (**26**) | *in vitro* | ATPase IC_50_ = 0.29 µM | exp. LD_50_ (mouse, oral) = 340 µmol/kg | PD | **A. Natterman & Cie GmbH** | Hearing loss | (Meotti et al., 2003; Chen et al., 2021) |
| Cepharantine (**27**) | *in silico*  *in vitro* | Score = -10.3 kcal/mol (AutoDock)  ATPase IC_50_ = 400 µM | exp. CC_50_ = 39.3 µM  exp. CC_50_ = 30.9 µM  LD_50_ (EPA) = 2415 mg/kg | NP | Stephania sinica  Stephania cepharantha  Other flowering plants | Recovery of immunological function | (Fan et al., 2020; White et al., 2020; Zhang et al., 2022) |
| Lumacaftor (**28**) | *in silico*   *in vitro* | Score = -11.1 kcal/mol (AutoDock)  ATPase IC_50_ = 300 µM | exp. CC_50_ = 314.5 µM  LD_50_ (EPA) = 2822 mg/kg | PD | ORKAMBI  **Vertex Pharmaceuticals**  Note: Combination of ivacaftor and lumacaftor | Cystic fibrosis | (White et al., 2020; Day et al., 2021) |
| Vapreotide (**29**) | *in silico  in vitro  in vivo* | Score = -9.84 kcal/mol (MOE)  Helicase IC_50_ = 10 µM  EC_50_ (Vero E6) = 3.98 µM | exp. CC_50_ > 100 µM  LD_50_ (EPA) = 110 mg/kg | PD | Sanvar  **Debiovision Inc.**  (Not FDA approved) | oesophageal variceal bleeding | (Borgio et al., 2020; Muturi et al., 2022) |
| Grazoprevir (**30**) | *in silico*  *in vitro  in vivo* | Score = -9.23 kcal/mol (AutoDock)  Helicase IC_50_ = 2.5 µM  EC_50_ (Vero E6) = 2.08 µM | exp. CC_50_ > 100 µM  LD_50_ (EPA) = 139 mg/kg | PD | Zepatier  **Merck**  Note: Combination of grazoprevir and elbasvir | Hepatitis C | (Gurung, 2020; Muturi et al., 2022) |
| Simeprevir (**31**) | *in silico  in vitro  in vivo* | Score = -10.42 kcal/mol (AutoDock)  Helicase IC_50_ = 1.25 µM  EC_50_ (Vero E6) = 1.41 µM | exp. CC_50_ = 32.71 µM  LD_50_ (EPA) = 41 mg/kg | PD | OLYSIO  **Janssen Therapeutics** | Hepatitis C | (Gurung, 2020; Muturi et al., 2022) |
| FPA124 (**32**) | *in vitro*  *in vivo* | Helicase IC_50_ = 8.5 µM  Helicase IC_50_ (+T20) = 8.4 µM  EC_50_ (Vero E6) = 14.0 µM | LC_50_ (Lazar) = 12.3 mg/kg/day  “FPA124 showed cytotoxicity at 100 µM” | SP |  |  | (Zeng et al., 2021) |
| Suramin (**33**) | *in vitro*  *in vivo* | Helicase IC_50_ = 0.94 µM  Helicase IC_50_ (+T20) = 1.1 µM  EC_50_ (Vero E6) = 9.90 µM | exp. LD_50_ (mouse, I.P.) = 750 mg/kg  LD_50_ (QSAR) = 2300 mg/kg/day | PD | GERMANIN  **Bayer** | African sleeping sickness and river blindness | (Balzarini et al., 1986; Zeng et al., 2021) |
| SSYA10-001 (**18**) | *in vitro*  *in vivo* | Helicase IC_50_ = 7.5 µM  Helicase IC_50_ (+T20) = 28 µM  EC_50_ (Vero E6) = 80.5 µM | exp. CC_50_ > 250 µM  LD_50_ (EPA) = 1198 mg/kg  LD_50_ (QSAR) = 120 mg/kg/day | SP |  |  | (Adedeji et al., 2012; Zeng et al., 2021) |
| SSYA10-001 (**18**) | *in vitro* | Helicase IC_50_ = 0.05 µM  ATPase IC_50_ = >3 µM | exp. CC_50_ > 250 µM  LD_50_ (EPA) = 1198 mg/kg  LD_50_ (QSAR) = 120 mg/kg/day | SP |  |  | (Adedeji et al., 2012; Corona et al., 2022) |
| Myricetin (**6**) | *in vitro*  *in vivo* | Helicase IC_50_ = 24 µM  Helicase IC_50_ (+T20) = 125 µM  EC_50_ (Vero E6) = 31.6 µM | exp. LD_50_ (Mouse, I.P.) = 1410 mg/kg  LD_50_ (EPA) = 1636 mg/kg | NP (flavonoid) | Many natural sources, e.g., tomatoes and oranges | Many reported effects, e.g., anti-oxidative and hypo-glycemic | (Zeng et al., 2021) |
| Myricetin (**6**) | *in vitro* | Helicase IC_50_ = 0.41 µM  ATPase IC_50_ = >30 µM | exp. LD_50_ (Mouse, I.P.) = 1410 mg/kg  LD_50_ (EPA) = 1636 mg/kg | NP (flavonoid) | Many natural sources, e.g., tomatoes and oranges | Many reported effects, e.g., anti-oxidative and hypo-glycemic | (Corona et al., 2022) |
| Quercetin (**8**) | *in vitro* | Helicase IC_50_ = 0.53 µM  ATPase IC_50_ = >30 µM | exp. LD_50_ (mouse, oral) = 160 mg/kg  LD_50_ (EPA) = 1606 mg/kg | NP (flavonoid) | Many natural sources, e.g., capers and kale | Many reported effects, e.g., anti-oxidative and antibacterial | (Lewis and Sax, 2004; Corona et al., 2022) |
| Kaempferol (**34**) | *in vitro* | Helicase IC_50_ = 0.76 µM  ATPase IC_50_ = >30 µM | exp. CC_50_ > 100 µM  LD_50_ (EPA) = 1686 mg/kg | NP (flavonoid) | *Lotus ucrainicus*  *Ardisia sanguinolenta* | Many reported effects, e.g., antibacterial and geroprotector | (Corona et al., 2022) |
| Flavanone (**35**) | *in vitro* | Helicase IC_50_ = 0.52 µM  ATPase IC_50_ = >30 µM | exp. CC_50_ > 100 µM  exp. LD_50_ (redwing, oral) = 75 mg/kg  LD_50_ (EPA) = 610 mg/kg | NP (flavonone) | *Betula pubescens* | Uterine cervical neoplasms (Kim et al., 2012) | (Schafer et al., 1983; Corona et al., 2022) |
| Licoflavone C (**36**) | *in vitro* | Helicase IC_50_ = 1.34 µM  ATPase IC_50_ = 24.6 µM | exp. CC_50_ > 100 µM  LD_50_ (EPA) = 501 mg/kg | NP (flavonoid) | *Genista ephedroides,*  *Artocarpus altilis* | Antibacterial and antifungal | (Edziri et al., 2012; Corona et al., 2022) |
| Zafirlukast (**37**) | *in silico*  *in vitro* | Score = -8.76 kcal/mol (MOE)  Helicase IC_50_ = 16.3 µM | exp. LD_50_ (mouse, oral) > 2000 mg/kg  LD_50_ (EPA) = 661 mg/kg | PD | ACCOLATE  **AstraZeneca** | asthma | (ACCOLATE^®^, 2013; Mehyar et al., 2021a) |
| Epirubicin HCl (**38**) | *in vitro* | Helicase IC_50_ = 0.31 µM | exp. LD_50_ (rat, oral) = 1350 mg/kg  LD_50_ (EPA) = 224 mg/kg | NP | *Streptomyces peucetius* |  | (Mehyar et al., 2021b) |
| Doxorubicin HCl (**39**) | *in vitro* | Helicase IC_50_ = 0.40 µM | exp. LD_50_ (mouse, oral) = 698 mg/kg  LD_50_ (EPA) = 224 mg/kg | PD | *Streptomyces peucetius* | Antibacterial and antineoplastic | (Mehyar et al., 2021b) |
| Daunorubicin HCl (**40**) | *in vitro* | Helicase IC_50_ = 0.46 µM | exp. LD_50_ (mouse, oral) = 205 mg/kg  LD_50_ (EPA) = 448 mg/kg | PD | *Streptomyces peucetius* | Antibacterial and antineoplastic | (Mehyar et al., 2021b) |
| Idarubicin HCl (**41**) | *in vitro* | Helicase IC_50_ = 0.72 µM | exp. LD_50_ (mouse, oral) = 14 mg/kg  LD_50_ (EPA) = 432 mg/kg | PD | **Hikma** | antineoplastic | (Mehyar et al., 2021b) |
| Mitoxantrone 2HCl (**42**) | *in vitro* | Helicase IC_50_ = 0.70 µM | exp. LD_50_ (mouse, oral) = 502 mg/kg  LD_50_ (EPA) = 967 mg/kg | PD | Novantrone  (Patent expired) | Antibacterial and antineoplastic | (Mehyar et al., 2021b) |

^a^ SMILES can be found in supplementary table 5. ^b^ Experimental (exp.) toxicity is referenced, predicted toxicity is displayed as: LD_50_ (mouse, oral) from a single injection after 48 hours predicted by US EPA TEST Software (Martin et al., 2008), LD_50_ (mouse, oral) predicted by QSAR Toolbox (Dimitrov et al., 2016), Maximum Recommended Daily Dose (MRDD) (human) predicted by the Lazar Web Server (Maunz et al., 2013) or LC_50_ (fathead minnow) predicted by Lazar Web Server (Maunz et al., 2013). ^c^ I.P. = intraperitoneal, I.V. = intravenous. ^d^ SP = Synthesized Product; NP = Natural product; PD = Pharmaceutical Drug. ^e^ Where possible taken from PubChem (Kim et al., 2019) and ChEBI (Hastings et al., 2016).


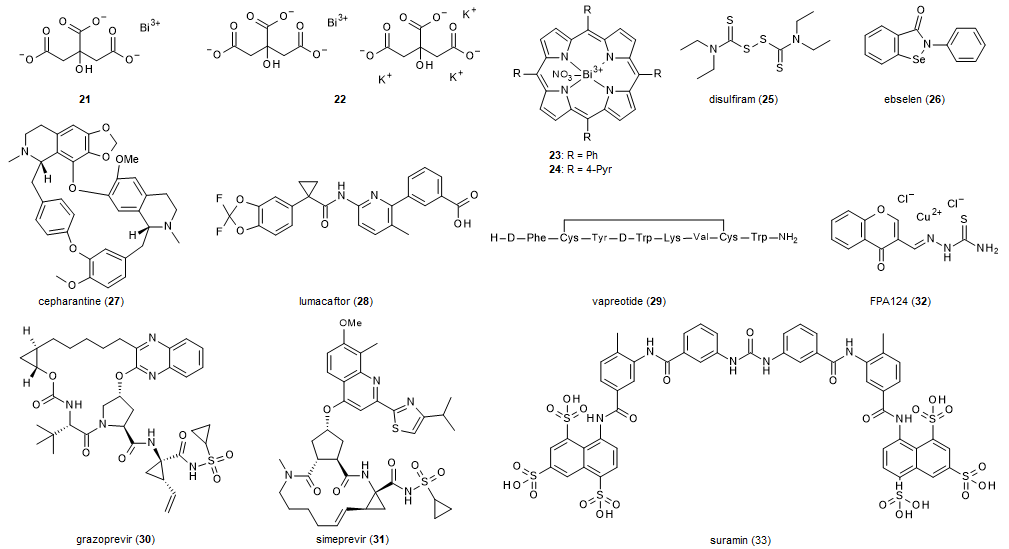


**Supplementary figure 2**: Chemical structures of SARS-CoV-2 inhibitors validated *in vitro*.


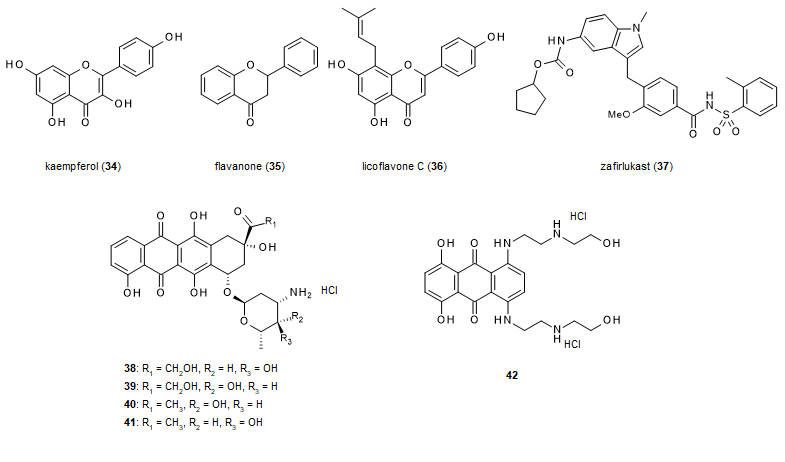


**Supplementary figure 2 (continued)**: Chemical structures of SARS-CoV-2 inhibitors validated *in vitro*.

**Supplementary table 3.** SARS-CoV-2 helicase virtual hits.

| **Name (#)^a^** | **Software** | **Activity / Score** | **Toxicity^b,c,e^** | **Classification^d^** | **Source^e^** | **Indication^e^** | **Reference** |
| --- | --- | --- | --- | --- | --- | --- | --- |
| Vapreotide (**29**) | MOE | Score = -9.84 kcal/mol | exp. CC_50_ > 100 µM  LD_50_ (EPA) = 110 mg/kg | PD | Sanvar  **Debiovision Inc.**  (Not FDA approved) | oesophageal variceal bleeding | (Borgio et al., 2020) |
| Atazanavir | MOE | Score = -9.32 kcal/mol | exp. CC_50_ = 312 µM  LD_50_ (EPA) = 852 mg/kg  LD_50_ (QSAR) = 2400 mg/kg/day | PD | Reyataz  **Bristol Meyers Squibb** | HIV/AIDS | (Borgio et al., 2020; Chaves et al., 2022) |
| Elbasvir | Glide | *No value provided* | exp. LD_50_ (mouse) > 1000 mg/kg  exp. NOAEL (rat, oral) = 1000mg/kg/day (Merck, 2022)  LD_50_ (EPA) = 112 mg/kg | PD | Zepatier  **Merck**  Note: Combination of grazoprevir and elbasvir | Hepatitis C | (Balasubramaniam and Shmookler Reis, 2020; Elbasvir: Formulation, 2022) |
| Daclatasvir | Glide | *No value provided* | exp. CC_50_ = 17-90 µM  LD_50_ (EPA) = 169 mg/kg  LD_50_ (QSAR) = 1000 mg/kg /day | PD | Daklinza  **Bristol Myers Squibb** | Hepatitis C | (Daklinza, 2014; Balasubramaniam and Shmookler Reis, 2020) |
| Astilbin | Glide  Desmond (MD) | Score = -6.24 kcal/mol  ΔG_binding_ = -90.99 kcal/mol (MM-GBSA) | exp. NOAEL (rat, oral) > 500 mg/kg/day  LD_50_ (EPA) = 2010 mg/kg | NP (flavonoid) | *Nicotiana Sylvestris*  *Hypericum perforatum*  *Smilar corbularia*  *Rhododendron simsii* | Anti-inflammatory, anti-bacterial and treatment of burns | (Moulari et al., 2006; Kimura et al., 2007; Gao et al., 2017; Naik et al., 2020) |
| Neoastilbin | Glide  Desmond (MD) | Score = -6.24 kcal/mol  ΔG_binding_ = -90.99 kcal/mol (MM-GBSA) | LD_50_ (EPA) = 2010 mg/kg | NP (flavonoid) | *Zalerion maritima*  *Smilax glabra*  *Neolitsea serícea*  *Dimorphandra mollis* |  | (Naik et al., 2020) |
| 5-hydroxy-7-(((2S,3R,4R,5R,6S)-3,4,5-trihydroxy-6-methyltetrahydro-2H-pyran-2-yl)oxy)-2-(3,4,5-trihydroxyphenyl)-4H-chromen-4-one | Glide  Desmond (MD) | Score = -5.69 kcal/mol  ΔG_binding_ = -87.42 kcal/mol (MM-GBSA) | LD_50_ (EPA) = 1716 mg/kg | NP (flavonoid) | *Bauhinia glauca* |  | (Naik et al., 2020) |
| methyl (2S,3S,4S,5R,6S)-6-[2-(3,4-dihydroxyphenyl)-5,7-dihydroxy-4-oxochromen-3-yl]oxy-3,4,5-trihydroxyoxane-2-carboxylate | Glide  Desmond (MD) | Score = -6.58 kcal/mol  ΔG_binding_ = -63.80 kcal/mol (MM-GBSA) | LD_50_ (EPA) = 1879 mg/kg | NP (flavonoid) | *Rumex obtusifolius* |  | (Naik et al., 2020) |
| Diplacol | Glide  Desmond (MD) | Score = -5.75 kcal/mol  ΔG_binding_ = -72.98 kcal/mol (MM-GBSA) | LD_50_ (EPA) = 1829 mg/kg | NP (flavonoid) | *Macaranga alnifolia*  *Diplacus aurantiacus* |  | (Naik et al., 2020) |
| Cangrelor | Glide | Score = -11.48 kcal/mol | LD_50_ (QSAR) = 280 mg/kg/day | PD | KENGREAL/ KENGREXAL  **Chiesi** | ischemia, clotting | (Ugurel et al., 2020) |
| Fludarabine | Glide | Score = -9.77 kcal/mol | exp. LD_50_ (mouse, I.P.) = 375 mg/kg  LD_50_ (EPA) = 2042 mg/kg  LD_50_ (QSAR) = 375 mg/kg/day | PD | Fludara  **Sanofi Genzyme**  Discontinued | Leukemia/lymphoma | (Ugurel et al., 2020) |
| Folic acid (Vitamin B_9_) | Glide | Score = 9.42 kcal/mol | exp. LD_50_ (mouse, oral) = 10000 mg/kg  exp. LD_50_ (mouse, I.P.) = 85 mg/kg  LD_50_ (EPA) = 2709 mg/kg  LD_50_ (QSAR) = 10000 mg/kg/day | NP / FD | Vegetables, beans, avocado, and some fruits | Birth defects | (Ugurel et al., 2020; Folic Acid, 2021) |
| Polydatin | Glide | Score = -7.28 kcal/mol | LD_50_ (EPA) = 3833 mg/kg  LD_50_ (QSAR) = 960 mg/kg/day | NP (stilbenoid glucoside) | *Polygonum cuspidatum*  *Picea sitchensis*  *Vitis rupestris* |  | (Ugurel et al., 2020) |
| Acarbose | Glide  GROMACS (MD) | Score = -10.73 kcal/mol | exp. LD_50_ (mouse, oral) = 24000 mg/kg  LD_50_ (EPA) = 12585 mg/kg | PD | Generic | Diabetes | (Sundar et al., 2021; Acarbose, 2021) |
| Quercetin 3-rhamnoside | Glide |  | exp. LD_50_ (mouse, I.P.) = 200 mg/kg  LD_50_ (EPA) = 1617 mg/kg | NP (Flavonoid glycoside) | *Zanthoxylum fagara*  *Xylopia emarginata*  *Lotus ucrainicus* |  | (James et al., 2022) |
| Acetyl cysteine | Glide  GROMACS (MD) | Score = -9.8 kcal/mol ΔG_binding_ = -27.9 kcal/mol (MM-PBSA) | exp. LD_50_ (rat, oral) = 5050 mg/kg  LD_50_ (EPA) = 2757 mg/kg | PD | Generic | Hepatic injury from acetaminophen overdose | (N-Acetyl-L-Cysteine, 2020; Pitsillou et al., 2022) |
| Clavulanic acid | Glide  GROMACS (MD) | Score = -8.0 kcal/mol  ΔG_binding_ = -27.2 kcal/mol (MM-PBSA) | exp. LD_50_ (mouse, oral) = 4526 mg/kg  LD_50_ (EPA) = 2520 mg/kg | NP | *Streptomyces clavuligerus* | Antibacterial | (Clavulanic Acid Potassium Salt, 2006; Pitsillou et al., 2022) |
| Homovanillic acid | Glide  GROMACS (MD) | Score = -8.9 kcal/mol  ΔG_binding_ = -27.2 kcal/mol (MM-PBSA) | LD_50_ (EPA) = 699 mg/kg | NP | Metabolite of dopamine  *Aloe Africana*  *Ginkgo biloba* |  | (Pitsillou et al., 2022) |
| Amentoflavone | Glide | Score = -9.8 kcal/mol | LD_50_ (EPA) = 1450.32 mg/kg | NP (biflavonoid) | *Cnestis ferruginea*  *Podocarpus elongatus*  *Callitris acuminata* | Antiviral  Depression | (Mbaveng et al., 2014; Hossain et al., 2022) |
| Glutathione | Glide  AutoDock | Score = -10.31 kcal/mol  Score = -6.4 kcal/mol | exp. LD_50_ (mouse, oral) = 5000 mg/kg  LD_50_ (EPA) = 1047 mg/kg | NP (peptide) | Endogenously produced in Homo sapiens | Anti-oxidative and nutraceutical | (Alanazi et al., 2022; L-glutathione reduced, 2022) |
| Nicotinamide adenine dinucleotide (NADH) | Glide  AutoDock | Score = -9.54 kcal/mol  Score = -10.5 kcal/mol | exp. NOAEL (beagle dogs, iv) = 500 mg/kg/day  LD_50_ (EPA) = 658 mg/kg | NP | Endogenously produced in Homo sapiens | Parkinson's disease, chronic fatigue syndrome, Alzheimer's disease and cardiovascular disease | (Eliseev and Marikhina, 1986; Birkmayer et al., 2004; Alanazi et al., 2022) |
| Quercetin-3-O-rutinoside | Glide  AutoDock | Score = -7.85 kcal/mol  Score = -7.1 kcal/mol | exp. LD_50_ (mouse, I.V.) = 950 mg/kg  LD_50_ (EPA) = 2469 mg/kg | NP (flavonoid glycoside) | *Prunus mume*  *Ruta graveolens* | Anti-oxidative | (Alanazi et al., 2022) |
| Posaconazole | AutoDock  AMBER16 | Score = -9.5 kcal/mol  ΔG_binding_ = -54.8 kcal/mol (MM-GBSA) | exp. LD_50_ (mouse, oral) > 3000 mg/kg  LD_50_ (EPA) = 247 mg/kg | FD | Noxafil/Posanol  **Merck**  (Generic since 2006) | Antifungal | (Abidi et al., 2021; Posaconazole Suspension Formulation, 2022) |
| N,N'-(carbonylbis(1,3-dioxoisoindoline-5,2-diyl))diisonicotinamide | AutoDock | Score = -10.9 kcal/mol | LD_50_ (EPA) = 1589 mg/kg | SP |  |  | (Mirza and Froeyen, 2020) |
| 2,5-bis(1,3-diphenyl-1H-pyrazol-4-yl)thiazolo[5,4-d]thiazole | AutoDock | Score = -10.6 kcal/mol | LD_50_ (EPA) = 140 mg/kg | SP |  |  | (Mirza and Froeyen, 2020) |
| (E)-1-hydroxy-4-((2-methyl-4-((4-methylphenyl)sulfonamido)phenyl)diazenyl)-N-phenyl-2-naphthamide | AutoDock | Score = -10.2 kcal/mol | LD_50_ (EPA) = 2299 mg/kg | SP |  |  | (Mirza and Froeyen, 2020) |
| 6,6'-(methylenebis(4,1-phenylene))bis(1H-furo[3,4-f]isoindole-1,3,5,7(6H)-tetraone) | AutoDock | Score = -10.2 kcal/mol | LD_50_ (EPA) = 490 mg/kg | SP |  |  | (Mirza and Froeyen, 2020) |
| Oxyphenisatin | AutoDock | Score = -9.1 kcal/mol | exp. LD_50_ (rat, oral) = 90 mg/kg  LD_50_ (EPA) = 1002 mg/kg | PD | Lavema  **Winthrop**  (Discontinued) | Laxative | (Oxyphenisatin, 2019; Iftikhar et al., 2020) |
| Meclonazepam | AutoDock | Score = -10.5 kcal/mol | LD_50_ (EPA) = 914 mg/kg | PD | **Hoffmann-La Roche**  (Off-market) | Sedative | (Iftikhar et al., 2020) |
| Tomentodiplacone B | AutoDock | Score = -8.4 kcal/mol | LD_50_ (EPA) = 2338 mg/kg | NP (flavonoid) | *Silybum Marianum*  *Paulownia tomentosa* | Antibacterial | (Smejkal et al., 2008; Kousar et al., 2020) |
| Osajin | AutoDock | Score = -8.2 kcal/mol | LD_50_ (EPA) = 413 mg/kg  LD_50_ (QSAR) = 560 mg/kg /day | NP (flavonoid) | *Erythrina varigatae*  *Deguelia hatschbachii*  *Euchresta japonica* | Prostate cancer | (Huang et al., 2019; Kousar et al., 2020) |
| 2-[2-(3,4-dihydroxyphenyl)-3-(hydroxymethyl)-2,3-dihydro-1,4-benzodioxin-6-yl]-3,5,7-trihydroxy-chroman-4-one | AutoDock | Score = -8.2 kcal/mol |  | NP | *Tanacetum parthenium* |  | (Kousar et al., 2020) |
| Rhamnetin | AutoDock | Score = -8.1 kcal/mol | LD_50_ (EPA) = 717 mg/kg  LD_50_ (QSAR) = 520 mg/kg/day | NP (flavonoid) | *Solanum nigrum*  *Ammannia auriculata*  *Chromolaena odorata* | Anti-oxidative and anti-inflammatory | (Kousar et al., 2020; Medeiros et al., 2022) |
| Silydianin | AutoDock | Score = -8.1 kcal/mol | LD_50_ (EPA) = 532mg/kg | NP (flavonoid) | *Silybum marianum*  *Silybum eburneum* | Anti-oxidative, anti-neoplastic and hepatoprotective | (Soleimani et al., 2019; Kousar et al., 2020) |
| Simeprevir (**31**) | AutoDock | Score = -10.42 kcal/mol | exp. CC_50_ = 32.71 µM  LD_50_ (EPA) = 41 mg/kg | PD | OLYSIO  **Janssen Therapeutics** | Hepatitis C | (Gurung, 2020; Muturi et al., 2022) |
| Paritaprevir | AutoDock | Score = -9.70 kcal/mol | LD_50_ (EPA) = 509 mg/kg | PD | Technivie/ Viekira  **ABBVIE INC**  (Discontinued) | Hepatitis C | (Gurung, 2020) |
| Grazoprevir (**30**) | AutoDock | Score = -9.23 kcal/mol | exp. CC_50_ > 100 µM  LD_50_ (EPA) = 139 mg/kg | PD | Zepatier  **Merck**  Note: Combination of grazoprevir and elbasvir | Hepatitis C | (Gurung, 2020; Muturi et al., 2022) |
| picrasidine M | AutoDock | Score = -10.5 kcal/mol | LD_50_ (EPA) = 236 mg/kg | NP (alkaloid) | *Picrasma Quassioides* |  | (Vivek-Ananth et al., 2021) |
| (+)-Epiexcelsin | AutoDock | Score = -9.0 kcal/mol | LD_50_ (EPA) = 104mg/kg | NP | *Litsea verticillate*  *Piper arborescens*  *Piper macropiper* |  | (Vivek-Ananth et al., 2021) |
| Isorhoeadine | AutoDock | Score = -8.9 kcal/mol | LD_50_ (EPA) = 145mg/kg | NP (alkaloid) | *Papaver rhoeas.*  *Papaver argemone*  *Papaver dubium* |  | (Vivek-Ananth et al., 2021) |
| Euphorbetin | AutoDock | Score = -8.9 kcal/mol | LD_50_ (EPA) = 1002 mg/kg | NP (flavonoid) | *Viola yedoensis Makino* |  | (Vivek-Ananth et al., 2021) |
| Picrasidine N | AutoDock | Score = -8.9 kcal/mol | LD_50_ (EPA) = 355mg/kg | NP (alkaloid) | *Picrasma Quassioides* | Metabolic regulation | (Zhao et al., 2016; Vivek-Ananth et al., 2021) |
| [(4aS,10bS)-5,5,8-trimethylspiro[2,4,4a,10b-tetrahydropyrano[3,2-c]chromene-3,3'-azetidine]-1'-yl]-[3-(1,2,4-triazol-4-yl)phenyl]methanone | AutoDock | Score = -10.2 kcal/mol | LD_50_ (EPA) = 697 mg/kg | SP |  |  | (Ahmad et al., 2021) |
| 2-methyl-6-(1-phenylethyl)phenol  (L2) | AutoDock | Score = -6.4 to -6.7 kcal/mol | LD_50_ (EPA) = 1359 mg/kg | SP |  |  | (Zia et al., 2021) |
| 2-amino-9-[rac-(4aR,6R,7R,7aR)-7-hydroxy-2-isopropoxy-2-oxo-7-propa-1,2-dienyl-4,4a,6,7a-tetrahydrofuro[3,2-d][1,3,2]dioxaphosphinin-6-yl]-3H-purin-6-one | AutoDock | Score = -9.6 kcal/mol | LD_50_ (EPA) = 222 mg/kg | SP |  |  | (García et al., 2021) |
| 5-[(4-chlorophenyl)-(6-hydroxy-4-oxo-2-thioxo-1H-pyrimidin-5-yl)methyl]-6-hydroxy-2-thioxo-1H-pyrimidin-4-one | AutoDock  GROMACS (MD) | Score = -12.4 kcal/mol  ΔG_binding_ = -328.6 kcal/mol (MM-PBSA) | LD_50_ (EPA) = 1727 mg/kg | SP |  |  | (El Hassab et al., 2022) |
| (**10**) | AutoDock | Score = -7.73 kcal/mol | LD_50_ (EPA) = 774 mg/kg | SP |  |  | (Perez-Lemus et al., 2022) |
| Bananin (4) | AutoDock | Score = -3.83 kcal/mol | exp. CC_50_ = 390 µM  LD_50_ (EPA) = 389 mg/kg | SP |  |  | (Tanner et al., 2005; Perez-Lemus et al., 2022) |
| SSYA10-001 (18) | AutoDock | Score = -7.83 kcal/mol | exp. CC_50_ > 250 µM  LD_50_ (EPA) = 1198 mg/kg  LD_50_ (QSAR) = 120 mg/kg/day | SP |  |  | (Adedeji et al., 2012; Perez-Lemus et al., 2022) |
| Ivermectin | AutoDock | Score = -10 kcal/mol | exp. LD_50_ (mouse, oral) = 11.6 mg/kg  LD_50_ (EPA) = 20 mg/kg | PD | Mectizan,  **Merck**  (Generic) | Anti-protozoal, anti-nematodal, and insecticide | (Mectizan, 1996; Samdani et al., 2022) |
| Scutellarein (9) | AutoDock | Score = -9.9 kcal/mol | LD_50_ (EPA) = 1308 mg/kg | NP (flavonoid) | Scutellaria baicalensis |  | (Samdani et al., 2022) |
| Myricetin (6) | AutoDock | Score = -9.7 kcal/mol | exp. LD_50_ (Mouse, I.P.) = 1410 mg/kg  LD_50_ (EPA) = 1636 mg/kg | NP (flavonoid) | Many natural sources, e.g., tomatoes and oranges | Many reported effects, e.g., anti-oxidative and hypo-glycemic | (Samdani et al., 2022) |
| Remdesivir | AutoDock | Score = -7.5 kcal/mol | LD_50_ (EPA) = 1837 mg/kg | PD | Veklury  **Gilead** | Antiviral (COVID-19) | (Bhargavi et al., 2022) |
| N-[(E)-[3-[(2-hydroxydibenzofuran-3-yl)amino]-1-methyl-3-oxo-propylidene]amino]-4-nitro-benzamide (WP3) | AutoDock | Score = -8.6 kcal/mol | LD_50_ (EPA) = 627 mg/kg | NP | *Withania somnifera*  *Phyllanthus emblica* |  | (Bhargavi et al., 2022) |
| Fostamatinib | Convolutional Neural Networks (CNN) | - | LD_50_ (EPA) = 195 mg/kg | PD | Tavalisse / Tavlesse  **Rigel Pharmaceuticals Inc.** | Chronic immune thrombocytopenia | (Azmoodeh et al., 2022) |
| Flavin adenine dinucleotide (FAD) | Convolutional Neural Networks (CNN) | - | LD_50_ (EPA) = 1589 mg/kg | NP | *Bacillus subtilis*  *Bremothecium ashbyi* | Eye disease | (Azmoodeh et al., 2022) |
| Miconazole | Convolutional Neural Networks (CNN) | - | exp. LD_50­_ (rat, oral) = 550 mg/kg  LD_50_ (EPA) = 1277 mg/kg | PD | Oravig  **Galt Pharms** | Antifungal | (Miconazole (free base), 2021; Azmoodeh et al., 2022) |
| Flavin mononucleotide (FMN) | Convolutional Neural Networks (CNN) | - | exp. LD_50_ (mouse, I.V.) = 365 mg/kg  LD_50_ (EPA) = 3151 mg/kg | NP | *Thalassiosira pseudonana*  *Arabidopsis thalania* | Vitamin B deficiency | (Azmoodeh et al., 2022) |

^a^ SMILES can be found in supplementary table 5. ^b^ Experimental (exp.) toxicity is referenced, predicted toxicity is displayed as: LD_50_ (mouse, oral) from a single injection after 48 hours predicted by US EPA TEST Software (Martin et al., 2008) or LD_50_ (mouse, oral) predicted by QSAR Toolbox (Dimitrov et al., 2016). ^c^ I.P. = intraperitoneal, I.V. = intravenous. ^d^ SP = Synthesized Product; NP = Natural product; PD = Pharmaceutical Drug. ^e^ Where possible taken from PubChem (Kim et al., 2019) and ChEBI (Hastings et al., 2016).

**Supplementary table 4.** clinical trials for COVID-19 of reported SARS-CoV-2 helicase inhibitors (next page).

| **Name (#)** | **ClinicalTrials.gov ID** | **Recruitment Status on 16/09/22** | **Application for COVID-19** |
| --- | --- | --- | --- |
| Bismuth Subsalicylate | NCT04811339 | recruiting | treatment |
| Quercetin (**8**) | NCT05037240 | completed | prevention |
|  | NCT04468139 | unknown | treatment |
|  | NCT04851821 | completed | treatment |
|  | NCT05130671 | completed | symptom management |
|  | NCT04861298 | completed | symptom management |
|  | NCT04377789 | completed | treatment |
|  | NCT04578158 | completed | treatment |
|  | NCT04603690 | completed | treatment |
|  | NCT04622865 | recruiting | treatment |
|  | NCT04536090 | not yet recruiting | treatment |
|  | NCT04844658 | completed | treatment |
| Plant extracts (containing flavonoids) | NCT04999098 | not yet recruiting | prevention |
|  | NCT04810728 | completed | treatment |
|  | NCT04487964 | completed | treatment |
|  | NCT05092503 | not yet recruiting | treatment |
|  | NCT04705753 | completed | treatment |
|  | NCT04800224 | completed | treatment |
|  | NCT04981314 | recruiting | treatment |
|  | NCT04480593 | completed | treatment |
|  | NCT04380870 | recruiting | treatment |
| Disulfiram (**25**) | NCT04594343 | completed | treatment |
|  | NCT04485130 | suspended | treatment |
| Ebselen (**26**) | NCT04483973 | enrolling by invitation | treatment |
| Rutinoside, glucoside | NCT05387252 | not yet recruiting | reduce vaccine side effects |
| Zafirlukast (**37**) | NCT04871828 | recruiting | treatment |
| Atazanavir | NCT04468087 | active, not recruiting | treatment |
|  | NCT04452565 | recruiting | treatment |
|  | NCT04459286 | terminated | treatment |
| Daclatasvir | NCT04535869 | unknown | treatment |
|  | NCT04773756 | completed | treatment |
|  | NCT04443725 | unknown | treatment |
|  | NCT04468087 | active, not recruiting | treatment |
|  | NCT04757272 | completed | prevention |
|  | NCT04532931 | completed | treatment |
|  | NCT04561063 | completed | treatment |
|  | NCT04729153 | not yet recruiting | treatment |
| Anticoagulant (including cangrelor) | NCT04518735 | completed | treatment |
| Folic acid (Vitamin B_9_) | NCT04751669 | not yet recruiting | symptom management |
|  | NCT04631536 | active, not recruiting | symptom management |
|  | NCT04828538 | active, not recruiting | treatment and prevention |
|  | NCT04813471 | recruiting | treatment |
|  | NCT04354428 | terminated | treatment |
|  | NCT04751669 | not yet recruiting | symptom management |
| Acetyl Cysteine | NCT04455243 | unknown | treatment |
|  | NCT04458298 | active, not recruiting | treatment |
|  | NCT04419025 | completed | symptom management |
|  | NCT04928495 | not yet recruiting | treatment |
|  | NCT05504655 | not yet recruiting | treatment |
|  | NCT04374461 | active, not recruiting | treatment |
|  | NCT04545008 | terminated | treatment |
|  | NCT04900129 | completed | treatment |
|  | NCT04792021 | completed | treatment |
|  | NCT04545008 | terminated | treatment |
| Glutathione | NCT05371288 | not yet recruiting | long-COVID-19 |
|  | NCT04742725 | completed | treatment |
|  | NCT04703036 | recruiting | treatment |
| Clavulanic acid | NCT04363060 | unknown | treatment |
| Nicotinamides | NCT04604704 | active, not recruiting | treatment of post-COVID-19 syndrome |
|  | NCT04809974 | recruiting | long-COVID-19 |
|  | NCT04910230 | completed | treatment |
|  | NCT04407390 | unknown | treatment |
| Posaconazole | NCT05065658 | recruiting | treatment |
| Ivermectin | *A total of 88 clinical trials are retrieved for search: “COVID-19”+ “ivermectin”* | | |
| Remdesivir | NCT04292899 | completed | treatment |
|  | NCT04292730 | completed | treatment |
|  | NCT04539262 | completed | treatment |
|  | NCT04409262 | completed | treatment |
|  | NCT04401579 | completed | treatment |
|  | *An additional 133 clinical trials are retrieved for search: “COVID-19”+ “remdesivir”* | | |
| Fostamatinib | NCT04579393 | completed | treatment |
|  | NCT04629703 | active, not recruiting | treatment |

**Supplementary table 5.** SMILES of compounds reported.

| Name / Number | SMILES |
| --- | --- |
| Molnupiravir | CC(C)C(=O)OC[C@H]1O[C@H]([C@H](O)[C@@H]1O)N2C=CC(=NC2=O)NO |
| Remdesivir | CCC(CC)COC(=O)[C@H](C)N[P@](=O)(OC[C@H]1O[C@](C#N)([C@H](O)[C@@H]1O)c2ccc3c(N)ncnn23)Oc4ccccc4 |
| ritonavir | CC(C)[C@H](NC(=O)N(C)Cc1csc(n1)C(C)C)C(=O)N[C@H](C[C@H](O)[C@H](Cc2ccccc2)NC(=O)OCc3cncs3)Cc4ccccc4 |
| nirmaltrelvir | CC(C)(C)[C@H](NC(=O)C(F)(F)F)C(=O)N1C[C@H]2[C@@H]([C@H]1C(=O)N[C@@H](C[C@@H]3CCNC3=O)C#N)C2(C)C |
| Vanilinbananin (1) | COc1cc(ccc1O)C23OC4(O)CC(O)(CC(O)(C4)O2)O3 |
| Iodobananin (2) | Cc1nc(I)c(C(=O)O)c(c1O)C23OC4(O)CC(O)(CC(O)(C4)O2)O3 |
| Eubananin (3) | COc1cc(CC(C)c2nc(C)c(O)c(c2CO)C34OC5(O)CC(O)(CC(O)(C5)O3)O4)ccc1O |
| Bananin (4) | Cc1ncc(CO)c(c1O)C23OC4(O)CC(O)(CC(O)(C4)O2)O3 |
| Ranitidine Bismuth Citrate (5) | [Bi+3].CN\C(=C/[N+](=O)[O-])\NCCSCc1oc(CN(C)C)cc1.OC(CC(=O)[O-])(CC(=O)[O-])C(=O)[O-] |
| Myricetin (6) | OC1=C(Oc2cc(O)cc(O)c2C1=O)c3cc(O)c(O)c(O)c3 |
| Baicalein (7) | OC1=C(Oc2cc(O)c(O)c(O)c2C1=O)c3ccccc3 |
| Quercetin (8) | OC1=C(Oc2cc(O)cc(O)c2C1=O)c3ccc(O)c(O)c3 |
| Scutellarein (9) | OC1=C(Oc2cc(O)c(O)c(O)c2C1=O)c3ccc(O)cc3 |
| (10) | COc1ccc(cc1O)C2=C(O)C(=O)c3c(O)cc(OCc4cccc(c4)C#N)cc3O2 |
| (11) | COc1ccc(cc1OC)C2=C(O)C(=O)c3c(O)cc(OCc4ccc(Cl)cc4)cc3O2 |
| (12) | OC1=C(Oc2cc(OCc3cccc(c3)C#N)cc(O)c2C1=O)c4ccc(O)c(O)c4 |
| (13) | OC1=C(Oc2cc(OCc3cccc(Cl)c3)cc(O)c2C1=O)c4ccc(O)c(O)c4 |
| (14) | OC1=C(Oc2cc(OCc3ccc(Cl)cc3)cc(O)c2C1=O)c4ccc(O)c(O)c4 |
| 6-(3-chloro-benzyloxy)-5-hydroxy-2-(3-iodo- benzyloxy)-chromen-4-one (15) | Oc1c(OCc2cccc(Cl)c2)ccc3OC(=CC(=O)c13)OCc4cccc(I)c4 |
| (Z)-4-(3-((4-chlorobenzyl)oxy) phenyl)-2-hydroxy-4-oxobut-2-enoic acid (16) | OC(=O)\C(=C\C(=O)c1cccc(OCc2ccc(Cl)cc2)c1)\O |
| HE602 (17) | Clc1ccc(NC(=O)c2cccc(OCc3ccccc3)c2)cc1c4oc5cccnc5n4 |
| SSYA10-001 (18) | [O-][N+](=O)c1ccccc1SCc2nnc(S)n2CC=C |
| (E)-3-(furan-2-yl)-N-(4-sulfamoyl phenyl)acrylamide (19) | NS(=O)(=O)c1ccc(NC(=O)\C=C\c2occc2)cc1 |
| 7-ethyl-8-mercapto-3-methyl-3,7-dihydro-1H-purine-2,6-dione (20) | CCN1C2C(N=C1S)N(C)C(=O)NC2=O |
| Bismuth Citrate (21) | [O-]C(CC(CC([O-])=O)(C([O-])=O)O)=O.[Bi+3] |
| Bismuth Potassium Citrate (22) | [O-]C(CC(CC([O-])=O)(C([O-])=O)O)=O.[O-]C(CC(CC([O-])=O)(C([O-])=O)O)=O.[K+].[K+].[K+].[Bi+3] |
| Bismuth(III) tetraphenylporphyrinate (23) | [O-][N+](O[Bi]1(n2c(C(c3ccccc3)=C3C=C4)ccc22)(N5=C6C=CC5=C2c2ccccc2)N3=C4C(c2ccccc2)=C(C=C2)N1C2=C6c1ccccc1)=O |
| Bismuth(III) tetra-4-pyridylporphyrinate (24) | [O-][N+](O[Bi]1(n2c(C(c3ccncc3)=C3C=C4)ccc22)(N5=C6C=CC5=C2c2ccncc2)N3=C4C(c2ccncc2)=C(C=C2)N1C2=C6c1ccncc1)=O |
| Disulfiram (25) | CCN(CC)C(=S)SSC(=S)N(CC)CC |
| Ebselen (26) | C1=CC=C(C=C1)N2C(=O)C3=CC=CC=C3[Se]2 |
| Cepharantine (27) | CN(CCc1c2)[C@H](Cc(cc3)cc(Oc4ccc(C[C@@H](c5c6O7)N(C)CCc5cc5c6OCO5)cc4)c3OC)c1cc7c2OC |
| Lumacaftor (28) | Cc(cc1)c(-c2cc(C(O)=O)ccc2)nc1NC(C1(CC1)c(cc1)cc(O2)c1OC2(F)F)=O |
| Vapreotide (29) | CC(C)[C@@H](C(N[C@@H](CSSC[C@@H](C(N[C@@H](Cc(cc1)ccc1O)C(N[C@H](Cc1c[nH]c2c1cccc2)C(N[C@H]1CCCCN)=O)=O)=O)NC([C@@H](Cc2ccccc2)N)=O)C(N[C@@H](Cc2c[nH]c3c2cccc3)C(N)=O)=O)=O)NC1=O |
| Grazoprevir (30) | CC(C)(C)[C@@H](C(N(C[C@@H](C1)Oc2nc3cc(OC)ccc3nc2CCCCC[C@H](C2)[C@@H]2O2)[C@@H]1C(N[C@](C1)([C@@H]1C=C)C(NS(C1CC1)(=O)=O)=O)=O)=O)NC2=O |
| Simeprevir (31) | O=C([C@]1(C[C@@]1([H])/C=C\CCCCN2C)NC([C@]3([H])C[C@@H](OC4=CC(C5=NC(C(C)C)=CS5)=NC6=C(C)C(OC)=CC=C46)C[C@@]3([H])C2=O)=O)NS(=O)(C7CC7)=O |
| FPA124 (32) | O=C(C(/C=N/NC(N)=S)=CO1)C2=C1C=CC=C2.[Cu+2].[Cl-].[Cl-] |
| Suramin (33) | CC1=C(C=C(C(NC2=C3C(C=C(S(O)(=O)=O)C=C3S(O)(=O)=O)=C(S(O)(=O)=O)C=C2)=O)C=C1)NC(C4=CC(NC(NC5=CC=CC(C(NC6=C(C=CC(C(NC7=C8C(C=C(S(O)(=O)=O)C=C8S(O)(=O)=O)=C(S(O)(=O)=O)C=C7)=O)=C6)C)=O)=C5)=O)=CC=C4)=O |
| Kaempferol (34) | O=c1c(O)c(-c2ccc(O)cc2)oc2cc(O)cc(O)c12 |
| Flavanone (35) | O=C1CC(Oc2ccccc12)c3ccccc3 |
| Licoflavone C (36) | CC(=CCC1=C2C(=C(C=C1O)O)C(=O)C=C(O2)C3=CC=C(C=C3)O)C |
| Zafirlukast (37) | Cc1ccccc1S(=O)(=O)NC(=O)c2cc(OC)c(cc2)Cc3cn(C)c4ccc(cc43)NC(=O)OC5CCCC5 |
| Epirubicin HCl (38) | C[C@H]1[C@@H]([C@H](C[C@@H](O1)O[C@H]2C[C@@](CC3=C2C(=C4C(=C3O)C(=O)C5=C(C4=O)C(=CC=C5)OC)O)(C(=O)CO)O)N)O.Cl |
| Doxorubicin HCl (39) | C[C@H]1[C@H]([C@H](C[C@@H](O1)O[C@H]2C[C@@](CC3=C2C(=C4C(=C3O)C(=O)C5=C(C4=O)C(=CC=C5)OC)O)(C(=O)CO)O)N)O.Cl |
| Daunorubicin HCl (40) | C[C@H]1[C@H]([C@H](C[C@@H](O1)O[C@H]2C[C@@](CC3=C2C(=C4C(=C3O)C(=O)C5=C(C4=O)C(=CC=C5)OC)O)(C(=O)C)O)N)O.Cl |
| Idarubicin HCl (42) | C[C@H]1[C@H]([C@H](C[C@@H](O1)O[C@H]2C[C@@](CC3=C2C(=C4C(=C3O)C(=O)C5=CC=CC=C5C4=O)O)(C(=O)C)O)N)O.Cl |
| Mitoxantrone 2HCl (41) | C1=CC(=C2C(=C1NCCNCCO)C(=O)C3=C(C=CC(=C3C2=O)O)O)NCCNCCO.Cl.Cl |
| Atazanavir | O=C(OC)N[C@H](C(=O)N[C@@H](Cc1ccccc1)[C@@H](O)CN(NC(=O)[C@@H](NC(=O)OC)C(C)(C)C)Cc3ccc(c2ncccc2)cc3)C(C)(C)C |
| Elbasvir | CC(C)[C@@H](C(=O)N1CCC[C@H]1c2[nH]cc(n2)c3ccc4c(c3)cc-5n4[C@@H](Oc6c5ccc(c6)c7c[nH]c(n7)[C@@H]8CCCN8C(=O)[C@H](C(C)C)NC(=O)OC)c9ccccc9)NC(=O)OC |
| Daclatasvir | CC(C)[C@@H](C(=O)N1CCC[C@H]1c2[nH]c(cn2)c3ccc(cc3)c4ccc(cc4)c5cnc([nH]5)[C@@H]6CCCN6C(=O)[C@H](C(C)C)NC(=O)OC)NC(=O)OC |
| Astilbin | C[C@@H]1O[C@@H](O[C@@H]2[C@H](Oc3cc(O)cc(O)c3C2=O)c4ccc(O)c(O)c4)[C@H](O)[C@H](O)[C@H]1O |
| Neoastilbin | C[C@@H]1O[C@@H](O[C@H]2[C@@H](Oc3cc(O)cc(O)c3C2=O)c4ccc(O)c(O)c4)[C@H](O)[C@H](O)[C@H]1O |
| 5-hydroxy-7-(((2S,3R,4R,5R,6S)-3,4,5-trihydroxy-6-methyltetrahydro-2H-pyran-2-yl)oxy)-2-(3,4,5-trihydroxyphenyl)-4H-chromen-4-one | C[C@@H]1O[C@@H](Oc2cc(O)c3C(=O)C=C(Oc3c2)c4cc(O)c(O)c(O)c4)[C@H](O)[C@H](O)[C@H]1O |
| methyl (2S,3S,4S,5R,6S)-6-[2-(3,4-dihydroxyphenyl)-5,7-dihydroxy-4-oxochromen-3-yl]oxy-3,4,5-trihydroxyoxane-2-carboxylate | COC(=O)[C@H]1O[C@@H](OC2=C(Oc3cc(O)cc(O)c3C2=O)c4ccc(O)c(O)c4)[C@H](O)[C@@H](O)[C@@H]1O |
| Diplacol | CC(=CCC/C(=C/CC1=C(C2=C(C=C1O)O[C@@H]([C@H](C2=O)O)C3=CC(=C(C=C3)O)O)O)/C)C |
| Cangrelor | CSCCNC1=NC(=NC2=C1N=CN2[C@H]3[C@@H]([C@@H]([C@H](O3)COP(=O)(O)OP(=O)(C(P(=O)(O)O)(Cl)Cl)O)O)O)SCCC(F)(F)F |
| Fludarabine | Fc1nc(c2ncn(c2n1)[C@@H]3O[C@@H]([C@@H](O)[C@@H]3O)CO)N |
| Folic acid (Vitamin B_9_) | n1c2C(=O)NC(N)=Nc2ncc1CNc3ccc(cc3)C(=O)N[C@H](C(O)=O)CCC(O)=O |
| Polydatin | OC1=CC(/C=C/C3=CC=C(O)C=C3)=CC(O[C@H]2[C@H](O)[C@@H](O)[C@H](O)[C@@H](CO)O2)=C1 |
| Acarbose | O([C@H]1[C@H](O)[C@@H](O)[C@H](O)O[C@@H]1CO)[C@H]4O[C@@H]([C@@H](O[C@H]3O[C@H](C)[C@@H](N[C@H]2/C=C(/CO)[C@@H](O)[C@H](O)[C@H]2O)[C@H](O)[C@H]3O)[C@H](O)[C@H]4O)CO |
| Quercetin 3-rhamnoside | C[C@H]1[C@@H]([C@H]([C@H]([C@@H](O1)OC2=C(OC3=CC(=CC(=C3C2=O)O)O)C4=CC(=C(C=C4)O)O)O)O)O |
| Acetyl cysteine | C/C(=N/[C@@H](CS)C(=O)O)/O |
| Clavulanic acid | O=C2N1[C@H](C(/O[C@@H]1C2)=C/CO)C(=O)O |
| Homovanillic acid | O=C(O)Cc1cc(OC)c(O)cc1 |
| Amentoflavone | O=C1\C=C(/Oc2cc(O)cc(O)c12)c6cc(c5c(O)cc(O)c3c5O/C(=C\C3=O)c4ccc(O)cc4)c(O)cc6 |
| Glutathione | C(CC(=O)N[C@@H](CS)C(=O)NCC(=O)O)[C@@H](C(=O)O)N |
| Nicotinamide adenine dinucleotide (NADH) | O=C(N)C1CC=C[N](C=1)[C@@H]2O[C@@H]([C@@H](O)[C@H]2O)COP([O-])(=O)OP(=O)([O-])OC[C@H]5O[C@@H](n4cnc3c(ncnc34)N)[C@H](O)[C@@H]5O |
| Quercetin-3-O-rutinoside | CC1C(C(C(C(O1)OCC2C(C(C(C(O2)OC3=C(OC4=CC(=CC(=C4C3=O)O)O)C5=CC(=C(C=C5)O)O)O)O)O)O)O)O |
| Posaconazole | O=C1N(/N=C\N1c2ccc(cc2)N7CCN(c6ccc(OCC3C[C@@](OC3)(c4ccc(F)cc4F)Cn5ncnc5)cc6)CC7)[C@@H](CC)[C@@H](O)C |
| N,N'-(carbonylbis(1,3-dioxoisoindoline-5,2-diyl))diisonicotinamide | O=C(NN1C(=O)c2ccc(cc2C1=O)C(=O)c3ccc4C(=O)N(NC(=O)c5ccncc5)C(=O)c4c3)c6ccncc6 |
| 2,5-bis(1,3-diphenyl-1H-pyrazol-4-yl)thiazolo[5,4-d]thiazole | c1ccc(cc1)c2nn(cc2c3nc4sc(nc4s3)c5cn(nc5c6ccccc6)c7ccccc7)c8ccccc8 |
| (E)-1-hydroxy-4-((2-methyl-4-((4-methylphenyl)sulfonamido)phenyl)diazenyl)-N-phenyl-2-naphthamide | Cc1ccc(cc1)S(=O)(=O)Nc2ccc(\N=N\c3cc(C(=O)Nc4ccccc4)c(O)c5ccccc35)c(C)c2 |
| 6,6'-(methylenebis(4,1-phenylene))bis(1H-furo[3,4-f]isoindole-1,3,5,7(6H)-tetraone) | O=C1OC(=O)c2cc3C(=O)N(C(=O)c3cc12)c4ccc(Cc5ccc(cc5)N6C(=O)c7cc8C(=O)OC(=O)c8cc7C6=O)cc4 |
| Oxyphenisatin | OC1=CC=C(C=C1)C1(C(=O)NC2=C1C=CC=C2)C1=CC=C(O)C=C1 |
| Meclonazepam | O=N(C1=CC2=C(NC([C@H](C)N=C2C3=CC=CC=C3)=O)C=C1)=O |
| Tomentodiplacone B | C/C(=C\CC1=C(C2=C(C=C1O)O[C@@H](CC2=O)C3=CC(=C(C=C3)O)OC)O)/C/C=C/C(C)(C)O |
| Osajin | CC(=CCc1c(c2c(=O)c(coc2c3c1OC(C=C3)(C)C)c4ccc(c(c4)O)O)O)C |
| Sesquiterpene Glycoside | OCC1Oc2cc(ccc2OC1c3ccc(O)c(O)c3)C4Oc5cc(O)cc(O)c5C(=O)C4O |
| Rhamnetin | COC1=CC(=C2C(=C1)OC(=C(C2=O)O)C3=CC(=C(C=C3)O)O)O |
| Silydianin | COC1=C(C=CC(=C1)C2[C@H]3CO[C@@]4([C@H]3C(=C[C@H]2C4=O)[C@@H]5[C@H](C(=O)C6=C(C=C(C=C6O5)O)O)O)O)O |
| Paritaprevir | Cc1cnc(cn1)C(=O)N[C@H]2CCCCC/C=C\[C@@H]3C[C@]3(NC(=O)[C@@H]4C[C@H](CN4C2=O)Oc5c6ccccc6c7ccccc7n5)C(=O)NS(=O)(=O)C8CC8 |
| picrasidine M | O=C1N2C3=CC=CC=C3C4=C2C(N(CCC5=NC=C(OC)C6=C5NC7=C6C=CC=C7OC)C=C4)=CC1=O |
| (+)-Epiexcelsin | COC1=C2OCOC2=CC([C@@H]3OC[C@@]4([H])[C@]3([H])CO[C@@H]4C5=CC(OC)=C(OCO6)C6=C5)=C1 |
| Isorhoeadine | CN1CCC2=CC3=C(OCO3)C=C2[C@]4([H])O[C@H](OC)C5=C(C=CC6=C5OCO6)[C@@]14[H] |
| Euphorbetin | O=C1C=CC2=C(O1)C=C(O)C(O)=C2C3=C(O)C(O)=CC(O4)=C3C=CC4=O |
| Picrasidine N | O=C1N2C3=CC=CC=C3C4=C2C(N(CCC5=NC=C(OC)C6=C5N(OC)C7=C6C=CC=C7)C=C4)=CC1=O |
| [(4aS,10bS)-5,5,8-trimethylspiro[2,4,4a,10b-tetrahydropyrano[3,2-c]chromene-3,3'-azetidine]-1'-yl]-[3-(1,2,4-triazol-4-yl)phenyl]methanone | CC1=CC2=C(C=C1)[C@@H]3[C@H](CC4(CN(C4)C(=O)C5=CC(=CC=C5)N6C=NN=C6)CO3)C(O2)(C)C |
| 2-methyl-6-(1-phenylethyl)phenol | CC(c1ccccc1)c2cccc(C)c2O |
| 2-amino-9-[rac-(4aR,6R,7R,7aR)-7-hydroxy-2-isopropoxy-2-oxo-7-propa-1,2-dienyl-4,4a,6,7a-tetrahydrofuro[3,2-d][1,3,2]dioxaphosphinin-6-yl]-3H-purin-6-one | CC(C)OP1(=O)OC[C@H]2O[C@@H](n3cnc4C(=O)N=C(N)Nc34)[C@@](O)(C=C=C)[C@@H]2O1 |
| 5-[(4-chlorophenyl)-(6-hydroxy-4-oxo-2-thioxo-1H-pyrimidin-5-yl)methyl]-6-hydroxy-2-thioxo-1H-pyrimidin-4-one | O=C1NC(NC(O)=C1C(C2=C(NC(NC2=O)=S)O)C3=CC=C(C=C3)Cl)=S |
| Ivermectin | CC[C@H](C)[C@@H]1[C@H](CC[C@@]2(O1)C[C@@H]3C[C@H](O2)C/C=C(/[C@H]([C@H](/C=C/C=C/4\CO[C@H]5[C@@]4([C@@H](C=C([C@H]5O)C)C(=O)O3)O)C)O[C@H]6C[C@@H]([C@H]([C@@H](O6)C)O[C@H]7C[C@@H]([C@H]([C@@H](O7)C)O)OC)OC)\C)C.C[C@H]1CC[C@]2(C[C@@H]3C[C@H](O2)C/C=C(/[C@H]([C@H](/C=C/C=C/4\CO[C@H]5[C@@]4([C@@H](C=C([C@H]5O)C)C(=O)O3)O)C)O[C@H]6C[C@@H]([C@H]([C@@H](O6)C)O[C@H]7C[C@@H]([C@H]([C@@H](O7)C)O)OC)OC)\C)O[C@@H]1C(C)C |
| N-[(E)-[3-[(2-hydroxydibenzofuran-3-yl)amino]-1-methyl-3-oxo-propylidene]amino]-4-nitro-benzamide | C\C(=N/NC(=O)c1ccc(cc1)[N+](=O)[O-])\CC(=O)Nc2cc3oc4ccccc4c3cc2O |
| Fostamatinib | Fc1cnc(nc1Nc2nc3N(C(=O)C(Oc3cc2)(C)C)COP(=O)(O)O)Nc4cc(OC)c(OC)c(OC)c4 |
| Flavin adenine dinucleotide (FAD) | c12cc(C)c(C)cc1N=C3C(=O)NC(=O)N=C3N2C[C@H](O)[C@H](O)[C@H](O)COP(=O)(O)OP(=O)(O)OC[C@@H]4[C@@H](O)[C@@H](O)[C@@H](O4)n5cnc6c5ncnc6N |
| Miconazole | Clc1cc(Cl)ccc1C(Cn2ccnc2)OCc3ccc(Cl)cc3Cl |
| Flavin mononucleotide (FMN) | Cc1cc2c(cc1C)n(c-3nc(=O)[nH]c(=O)c3n2)C[C@@H]([C@@H]([C@@H](COP(=O)(O)O)O)O)O |

**References**

Abidi, S. H., Almansour, N. M., Amerzhanov, D., Allemailem, K. S., Rafaqat, W., Ibrahim, M. A. A., et al. (2021). Repurposing potential of posaconazole and grazoprevir as inhibitors of SARS-CoV-2 helicase. *Sci. Rep.* 11, 10290. doi: 10.1038/s41598-021-89724-0.

Acarbose; SDS No. 11885 [Online] (2021). Available at: https://cdn.caymanchem.com/cdn/msds/11885m.pdf [Accessed August 17, 2022].

Adedeji, A. O., Singh, K., Calcaterra, N. E., DeDiego, M. L., Enjuanes, L., Weiss, S., et al. (2012). Severe Acute Respiratory Syndrome Coronavirus Replication Inhibitor That Interferes with the Nucleic Acid Unwinding of the Viral Helicase. *Antimicrob. Agents Chemother.* 56, 4718–4728. doi: 10.1128/AAC.00957-12.

Adedeji, A. O., Singh, K., Kassim, A., Coleman, C. M., Elliott, R., Weiss, S. R., et al. (2014). Evaluation of SSYA10-001 as a Replication Inhibitor of Severe Acute Respiratory Syndrome, Mouse Hepatitis, and Middle East Respiratory Syndrome Coronaviruses. *Antimicrob. Agents Chemother.* 58, 4894–4898. doi: 10.1128/AAC.02994-14.

Ahmad, S., Waheed, Y., Ismail, S., Bhatti, S., Abbasi, S. W., and Muhammad, K. (2021). Structure-Based Virtual Screening Identifies Multiple Stable Binding Sites at the RecA Domains of SARS-CoV-2 Helicase Enzyme. *Molecules* 26, 1446. doi: 10.3390/molecules26051446.

Alanazi, K. M., Farah, M. A., and Hor, Y.-Y. (2022). Multi-Targeted Approaches and Drug Repurposing Reveal Possible SARS-CoV-2 Inhibitors. *Vaccines* 10, 24. doi: 10.3390/vaccines10010024.

ACCOLATE^®^; Product monograph [Online] (2013). Available at: https://pdf.hres.ca/dpd_pm/00022728.PDF [accessed August 16, 2022]

Azmoodeh, S. K., Tsigelny, I. F., and Kouznetsova, V. L. (2022). Potential SARS-CoV-2 nonstructural proteins inhibitors: drugs repurposing with drug-target networks and deep learning. *Front. Biosci. (Landmark Ed.)* 27, 113. doi: 10.31083/j.fbl2704113.

Balasubramaniam, M., and Shmookler Reis, R. (2020). Computational Target-Based Drug Repurposing of Elbasvir, an Antiviral Drug Predicted to Bind Multiple SARS-CoV-2 Proteins. *ChemRxiv* [Preprint]. Available at: 10.26434/chemrxiv.12084822.v2.

Balzarini, J., Mitsuya, H., De Clercq, E., and Broder, S. (1986). Aurintricarboxylic acid and evans blue represent two different classes of anionic compounds which selectively inhibit the cytopathogenicity of human T-cell lymphotropic virus type III/lymphadenopathy-associated virus. *Biochem. Biophys. Res. Commun.* 136, 64–71. doi: 10.1016/0006-291X(86)90877-6.

Bhargavi, S., Madhan Shankar, S. R., and Jemmy, C. H. (2022). In silico and in vitro studies on inhibitors for SARS-CoV-2 non-structural proteins with dual herbal combination of Withania somnifera with five rasayana herbs. *J. Biomol. Struct. Dyn.,* 1–16. doi: 10.1080/07391102.2022.2046642.

Birkmayer, J. G. D., Nadlinger, K. F. R., and Hallström, S. (2004). On the safety of reduced nicotinamide adenine dinucleotide (NADH*). J. Environ. Pathol. Toxicol. Oncol.* 23, 179–194. doi: 10.1615/jenvpathtoxoncol.v23.i3.20.

Borgio, J. F., Alsuwat, H. S., Otaibi, W. M. A., Ibrahim, A. M., Almandil, N. B., Asoom, L. I. A., et al. (2020). State-of-the-art tools unveil potent drug targets amongst clinically approved drugs to inhibit helicase in SARS-CoV-2. *Arch. Med. Sci.* 16, 508–518. doi: 10.5114/aoms.2020.94567.

Chaves, O. A., Sacramento, C. Q., Ferreira, A. C., Mattos, M., Fintelman-Rodrigues, N., Temerozo, J. R., et al. (2022). Atazanavir Is a Competitive Inhibitor of SARS-CoV-2 Mpro, Impairing Variants Replication In Vitro and In Vivo. *Pharmaceuticals* 15, 21. doi: 10.3390/ph15010021.

Chen, T., Fei, C.-Y., Chen, Y.-P., Sargsyan, K., Chang, C.-P., Yuan, H. S., et al. (2021). Synergistic Inhibition of SARS-CoV-2 Replication Using Disulfiram/Ebselen and Remdesivir. *ACS Pharmacol. Transl. Sci.* 4, 898–907. doi: 10.1021/acsptsci.1c00022.

Cho, J.-B., Lee, J.-M., Ahn, H.-C., Jeong, Y.-J. (2015). Identification of a Novel Small Molecule Inhibitor Against SARS Coronavirus Helicase. *J. Microbiol. Biotechnol.* 25, 2007–2010. doi: 10.4014/jmb.1507.07078.

Clavulanic Acid Potassium Salt; SDS sc-207446 [Online] (2006). Available at: https://datasheets.scbt.com/sc-207446.pdf [Accessed August 17, 2022].

Corona, A., Wycisk, K., Talarico, C., Manelfi, C., Milia, J., Cannalire, R., et al. (2022). Natural Compounds Inhibit SARS-CoV-2 nsp13 Unwinding and ATPase Enzyme Activities. *ACS Pharmacol. Transl. Sci*. 5, 226–239. doi: 10.1021/acsptsci.1c00253.

Daklinza; Assessment report EMA/419836/2014 [Online] (2014). European Medicines Agency Available at: https://www.ema.europa.eu/en/documents/assessment-report/daklinza-epar-public-assessment-report_en.pdf [Accessed August 17, 2022].

Day, C. J., Bailly, B., Guillon, P., Dirr, L., Jen, F. E.-C., Spillings, B. L., et al. (2021). Multidisciplinary Approaches Identify Compounds that Bind to Human ACE2 or SARS-CoV-2 Spike Protein as Candidates to Block SARS-CoV-2-ACE2 Receptor Interactions. *mBio* 12, e03681-20. doi: 10.1128/mBio.03681-20.

Dimitrov, S. D., Diderich, R., Sobanski, T., Pavlov, T. S., Chankov, G. V., Chapkanov, A. S., et al. (2016). QSAR Toolbox - workflow and major functionalities. *SAR QSAR Environ. Res.* 27, 203–219. doi: 10.1080/1062936X.2015.1136680.

Edziri, H., Mastouri, M., Mahjoub, M. A., Mighri, Z., Mahjoub, A., and Verschaeve, L. (2012). Antibacterial, antifungal and cytotoxic activities of two flavonoids from Retama raetam flowers. *Molecules* 17, 7284–7293. doi: 10.3390/molecules17067284.

El Hassab, M. A., Eldehna, W. M., Al-Rashood, S. T., Alharbi, A., Eskandrani, R. O., Alkahtani, H. M., et al. (2022). Multi-stage structure-based virtual screening approach towards identification of potential SARS-CoV-2 NSP13 helicase inhibitors. *J. Enzyme Inhib. Med. Chem.* 37, 563–572. doi: 10.1080/14756366.2021.2022659.

Elbasvir: Formulation; SDS No. 530934-00017 [Online] (2022). Available at: https://www.msd.com/docs/product/safety-data-sheets/hh-sds/Elbasvir%20Formulation_HH_IN_6N.pdf [Accessed August 17, 2022].

Eliseev, V. V., and Marikhina, B. L. (1986). Comparative study of antihypoxic properties of some nucleosides and nucleotides. *Pharm. Chem. J.* 20, 160–162. doi: 10.1007/BF00758559.

Fan, H.-H., Wang, L.-Q., Liu, W.-L., An, X.-P., Liu, Z.-D., He, X.-Q., et al. (2020). Repurposing of clinically approved drugs for treatment of coronavirus disease 2019 in a 2019-novel coronavirus-related coronavirus model. *Chin. Med. J.* 133, 1051–1056. doi: 10.1097/CM9.0000000000000797.

Folic Acid; SDS No. 20515 [Online] (2021). Available at: https://cdn.caymanchem.com/cdn/msds/20515m.pdf [Accessed August 17, 2022].

Gao, Y., Li, C., Wang, Y., Liu, Y., Li, G., Fan, X., et al. (2017). Nonclinical safety of astilbin: A 4-week oral toxicity study in rats with genotoxicity, chromosomal aberration, and mammalian micronucleus tests. *Food Chem. Toxicol.* 107, 1–9. doi: 10.1016/j.fct.2017.06.024.

García, R., Hussain, A., Koduru, P., Atis, M., Wilson, K., Park, J. Y., et al. (2021). Identification of potential antiviral compounds against SARS-CoV-2 structural and non structural protein targets: A pharmacoinformatics study of the CAS COVID-19 dataset. *Comput. Biol. Med.* 133, 104364. doi: 10.1016/j.compbiomed.2021.104364.

Gurung, A. B. (2020). In silico structure modelling of SARS-CoV-2 Nsp13 helicase and Nsp14 and repurposing of FDA approved antiviral drugs as dual inhibitors. *Gene Rep*. 21, 100860. doi: 10.1016/j.genrep.2020.100860.

Hastings, J., Owen, G., Dekker, A., Ennis, M., Kale, N., Muthukrishnan, V., et al. (2016). ChEBI in 2016: Improved services and an expanding collection of metabolites. *Nucleic Acids Res.* 44, D1214-9. doi: 10.1093/nar/gkv1031.

Hossain, R., Sarkar, C., Hassan, S. M. H., Khan, R. A., Arman, M., Ray, P., et al. (2022). In Silico Screening of Natural Products as Potential Inhibitors of SARS-CoV-2 Using Molecular Docking Simulation. *Chin. J. Integr. Med.* 249–256.

Park, H. R., Yoon, H., Kim, M. K., Lee, S. D., Chong, Y. (2012). Synthesis and antiviral evaluation of 7-O-arylmethylquercetin derivatives against SARS-associated coronavirus (SCV) and hepatitis C virus (HCV). *Arch. Pharm. Res.* 35, 77–85. doi: 10.1007/s12272-012-0108-9.

Huang, S.-Y., Huang, G.-J., Hsieh, P.-F., Wu, H.-C., and Huang, W.-C. (2019). Osajin displays potential antiprostate cancer efficacy via impairment of fatty acid synthase and androgen receptor expression. *Prostate* 79, 1543–1552. doi: 10.1002/pros.23876.

Iftikhar, H., Ali, H. N., Farooq, S., Naveed, H., and Shahzad-ul-Hussan, S. (2020). Identification of potential inhibitors of three key enzymes of SARS-CoV2 using computational approach. *Comput. Biol. Med.* 122, 103848. doi: 10.1016/j.compbiomed.2020.103848.

James, J. P., Jyothi, D., and Priya, S. (2022). In silico Screening of Phytoconstituents with Antiviral Activities Against SARS-COV-2 Main Protease, Nsp12 Polymerase, and Nsp13 Helicase Proteins. *Lett. Drug Des. Discov.* 18, 841–857.

Kao, R. Y., Tsui, W. H. W., Lee, T. S. W., Tanner, J. A., Watt, R. M., Huang, J.-D., et al. (2004). Identification of novel small-molecule inhibitors of severe acute respiratory syndrome-associated coronavirus by chemical genetics. *Chem. Biol.* 11, 1293–1299. doi: 10.1016/j.chembiol.2004.07.013.

Keum, Y. S., Lee, J. M., Yu, M. S., Chin, Y. W., and Jeong, Y. J. (2013). Inhibition of SARS coronavirus helicase by baicalein. *Bull. Korean Chem. Soc.* 34. doi: 10.5012/bkcs.2013.34.11.3187.

Kim, J.-H., Kang, J. W., Kim, M. S., Bak, Y., Park, Y. S., Jung, K.-Y., et al. (2012). The apoptotic effects of the flavonoid N101-2 in human cervical cancer cells. *Toxicol. In Vitro* 26, 67–73. doi: 10.1016/j.tiv.2011.10.012.

Kim, M. K., Yu, M.-S., Park, H. R., Kim, K. B., Lee, C., Cho, S. Y., et al. (2011). 2,6-Bis-arylmethyloxy-5-hydroxychromones with antiviral activity against both hepatitis C virus (HCV) and SARS-associated coronavirus (SCV). *Eur. J. Med. Chem.* 46, 5698–5704. doi: 10.1016/j.ejmech.2011.09.005.

Kim, S., Chen, J., Cheng, T., Gindulyte, A., He, J., He, S., et al. (2019). PubChem 2019 update: improved access to chemical data. *Nucleic Acids Res.* 47, D1102–D1109. doi: 10.1093/nar/gky1033.

Kimura, Y., Sumiyoshi, M., and Sakanaka, M. (2007). Effects of Astilbe thunbergii rhizomes on wound healing: Part 1. Isolation of promotional effectors from Astilbe thunbergii rhizomes on burn wound healing. *J. Ethnopharmacol.* 109, 72–77. doi: 10.1016/j.jep.2006.07.007.

Kousar, K., Majeed, A., Yasmin, F., Hussain, W., and Rasool, N. (2020). Phytochemicals from Selective Plants Have Promising Potential against SARS-CoV-2: Investigation and Corroboration through Molecular Docking, MD Simulations, and Quantum Computations. *BioMed Res. Int.* 2020, e6237160. doi: 10.1155/2020/6237160.

Lee, C., Lee, J. M., Lee, N.-R., Jin, B.-S., Jang, K. J., Kim, D.-E., et al. (2009a). Aryl diketoacids (ADK) selectively inhibit duplex DNA-unwinding activity of SARS coronavirus NTPase/helicase. *Bioorg. Med. Chem. Lett.* 19, 1636–1638. doi: 10.1016/j.bmcl.2009.02.010.

Lee, C., Lee, J. M., Lee, N.-R., Kim, D.-E., Jeong, Y.-J., and Chong, Y. (2009b). Investigation of the pharmacophore space of Severe Acute Respiratory Syndrome coronavirus (SARS-CoV) NTPase/helicase by dihydroxychromone derivatives. *Bioorg. Med. Chem. Lett.* 19, 4538–4541. doi: 10.1016/j.bmcl.2009.07.009.

Lee, J.-M., Cho, J.-B., Ahn, H.-C., Jung, W., and Jeong, Y.-J. (2017). A Novel Chemical Compound for Inhibition of SARS Coronavirus Helicase. *J. Microbiol. Biotechnol.* 27, 2070–2073. doi: 10.4014/jmb.1707.07073.

Lewis, R. J., and Sax, N. I. (2004). Sax’s dangerous properties of industrial materials. Hoboken, N.J.: J. Wiley & Sons Available at: https://openlibrary.org/books/OL15569894M [Accessed August 8, 2022].

L-glutathione reduced; SDS No. 10007461 [Online] (2022). Available at: https://cdn.caymanchem.com/cdn/msds/10007461m.pdf [Accessed August 17, 2022].

Li, H.-Y., Hu, J., Zhao, S., Yuan, Z.-Y., Wan, H.-J., Lei, F., et al. (2012). Comparative Study of the Effect of Baicalin and Its Natural Analogs on Neurons with Oxygen and Glucose Deprivation Involving Innate Immune Reaction of TLR2/TNF 𝛼. *J. Biomed. Biotechnol.* 2012, e267890. doi: 10.1155/2012/267890.

Li, M., Shi, A., Pang, H., Xue, W., Li, Y., Cao, G., et al. (2014). Safety, tolerability, and pharmacokinetics of a single ascending dose of baicalein chewable tablets in healthy subjects. *J. Ethnopharmacol*. 156, 210–215. doi: 10.1016/j.jep.2014.08.031.

Martin, T. M., Harten, P., Venkatapathy, R., Das, S., and Young, D. M. (2008). A hierarchical clustering methodology for the estimation of toxicity. *Toxicol. Mech. Methods* 18, 251–266. doi: 10.1080/15376510701857353.

Maunz, A., Gütlein, M., Rautenberg, M., Vorgrimmler, D., Gebele, D., and Helma, C. (2013). lazar: a modular predictive toxicology framework. *Front. Pharmacol*. 4, 38. doi: 10.3389/fphar.2013.00038.

Mbaveng, A. T., Zhao, Q., and Kuete, V. (2014). “20 - Harmful and Protective Effects of Phenolic Compounds from African Medicinal Plants,” in Toxicological Survey of African Medicinal Plants, ed. V. Kuete (Elsevier), 577–609. doi: 10.1016/B978-0-12-800018-2.00020-0.

Mectizan; Approval Package 050742 [Online] (1996). Merck Research Laboratories Available at: https://www.accessdata.fda.gov/drugsatfda_docs/nda/96/050742ap.pdf [Accessed August 17, 2022].

Medeiros, D. L., Lima, E. T. G., Silva, J. C., Medeiros, M. A., and Pinheiro, E. B. F. (2022). Rhamnetin: a review of its pharmacology and toxicity. *J. Pharm. Pharmacol.* 74, 793–799. doi: 10.1093/jpp/rgab163.

Mehyar, N., Mashhour, A., Islam, I., Alhadrami, H. A., Tolah, A. M., Alghanem, B., et al. (2021a). Discovery of Zafirlukast as a novel SARS-CoV-2 helicase inhibitor using in silico modelling and a FRET-based assay. *SAR QSAR Environ. Res.* 32, 963–983. doi: 10.1080/1062936X.2021.1993995.

Mehyar, N., Mashhour, A., Islam, I., Gul, S., Adedeji, A. O., Askar, A. S., et al. (2021b). Using in silico modelling and FRET-based assays in the discovery of novel FDA-approved drugs as inhibitors of MERS-CoV helicase. *SAR QSAR Environ. Res.* 32, 51–70. doi: 10.1080/1062936X.2020.1857437.

Meotti, F. C., Borges, V. C., Zeni, G., Rocha, J. B. T., and Nogueira, C. W. (2003). Potential renal and hepatic toxicity of diphenyl diselenide, diphenyl ditelluride and Ebselen for rats and mice. *Toxicol. Lett.* 143, 9–16. doi: 10.1016/s0378-4274(03)00090-0.

Merck (2022). Elbasivir Formulation Safety Data Sheet. Available at: https://www.merck.com/docs/product/safety-data-sheets/hh-sds/Elbasvir%20Formulation_HH_US_EN.pdf.

Miconazole (free base); SDS BP2668-1 [Online] (2021). Available at: https://www.fishersci.com/store/msds?partNumber=BP26681&productDescription=MICONAZOLE+1G&vendorId=VN00033897&countryCode=US&language=en [Accessed August 17, 2022].

Mirza, M. U., and Froeyen, M. (2020). Structural elucidation of SARS-CoV-2 vital proteins: Computational methods reveal potential drug candidates against main protease, Nsp12 polymerase and Nsp13 helicase. *J. Pharm. Anal.* 10, 320–328. doi: 10.1016/j.jpha.2020.04.008.

Moulari, B., Pellequer, Y., Lboutounne, H., Girard, C., Chaumont, J.-P., Millet, J., et al. (2006). Isolation and in vitro antibacterial activity of astilbin, the bioactive flavanone from the leaves of Harungana madagascariensis Lam. ex Poir. (Hypericaceae). *J. Ethnopharmacol*. 106, 272–278. doi: 10.1016/j.jep.2006.01.008.

Muturi, E., Hong, W., Li, J., Yang, W., He, J., Wei, H., et al. (2022). Effects of simeprevir on the replication of SARS-CoV-2 in vitro and in transgenic hACE2 mice. *Int. J. Antimicrob. Agents* 59, 106499. doi: 10.1016/j.ijantimicag.2021.106499.

N-Acetyl-L-Cysteine; SDS ACR16028 [Online] (2020). Available at: https://www.fishersci.no/store/msds?partNumber=10475365&productDescription=1KG+N-Acetyl-L-cysteine%2C+98%25&countryCode=NO&language=en [Accessed August 17, 2022].

Naik, B., Gupta, N., Ojha, R., Singh, S., Prajapati, V. K., and Prusty, D. (2020). High throughput virtual screening reveals SARS-CoV-2 multi-target binding natural compounds to lead instant therapy for COVID-19 treatment. *Int. J. Biol. Macromol.* 160, 1–17. doi: 10.1016/j.ijbiomac.2020.05.184.

Oxyphenisatin; SDS FO65118 [Online] (2019). Available at: https://static.cymitquimica.com/products/3D/pdf/sds-FO65118.pdf [Accessed August 17, 2022].

Perez-Lemus, G. R., Menéndez, C. A., Alvarado, W., Byléhn, F., and de Pablo, J. J. (2022). Toward wide-spectrum antivirals against coronaviruses: Molecular characterization of SARS-CoV-2 NSP13 helicase inhibitors. *Sci. Adv.* 8, eabj4526. doi: 10.1126/sciadv.abj4526

Pitsillou, E., Liang, J., Hung, A., and Karagiannis, T. C. (2022). The SARS-CoV-2 helicase as a target for antiviral therapy: Identification of potential small molecule inhibitors by in silico modelling. *J. Mol. Graph. Model.* 114, 108193. doi: 10.1016/j.jmgm.2022.108193.

Posaconazole Suspension Formulation; SDS No. 28760-00018 [Online] (2022). Available at: https://www.merck.com/docs/product/safety-data-sheets/hh-sds/Posaconazole%20Suspension%20Formulation_HH_ID_6N.pdf [Accessed August 17, 2022].

Samdani, Md. N., Morshed, N., Reza, R., Asaduzzaman, M., and Islam, A. B. M. Md. K. (2022). Targeting SARS-CoV-2 non-structural protein 13 via helicase-inhibitor-repurposing and non-structural protein 16 through pharmacophore-based screening. *Mol. Divers.* doi: 10.1007/s11030-022-10468-8.

Schafer, E. W., Bowles, W. A., and Hurlbut, J. (1983). The acute oral toxicity, repellency, and hazard potential of 998 chemicals to one or more species of wild and domestic birds. *Arch. Environ. Contam. Toxicol.* 12, 355–382. doi: 10.1007/bf01059413.

Shu, T., Huang, M., Wu, D., Ren, Y., Zhang, X., Han, Y., et al. (2020). SARS-Coronavirus-2 Nsp13 Possesses NTPase and RNA Helicase Activities That Can Be Inhibited by Bismuth Salts. *Virol. Sin.* 35, 321–329. doi: 10.1007/s12250-020-00242-1.

Soleimani, V., Delghandi, P. S., Moallem, S. A., and Karimi, G. (2019). Safety and toxicity of silymarin, the major constituent of milk thistle extract: An updated review. *Phytother. Res.* 33, 1627–1638. doi: 10.1002/ptr.6361.

Sundar, S., Thangamani, L., Piramanayagam, S., Rahul, C. N., Aiswarya, N., Sekar, K., et al. (2021). Screening of FDA-approved compound library identifies potential small-molecule inhibitors of SARS-CoV-2 non-structural proteins NSP1, NSP4, NSP6 and NSP13: molecular modeling and molecular dynamics studies. *J. Proteins Proteom.* 12, 161–175. doi: 10.1007/s42485-021-00067-w.

Tanner, J. A., Zheng, B.-J., Zhou, J., Watt, R. M., Jiang, J.-Q., Wong, K.-L., et al. (2005). The Adamantane-Derived Bananins Are Potent Inhibitors of the Helicase Activities and Replication of SARS Coronavirus. *Chem. Biol.* 12, 303–311. doi: 10.1016/j.chembiol.2005.01.006.

Ugurel, O. M., Mutlu, O., Sariyer, E., Kocer, S., Ugurel, E., Inci, T. G., et al. (2020). Evaluation of the potency of FDA-approved drugs on wild type and mutant SARS-CoV-2 helicase (Nsp13). *Int. J. Biol. Macromol.* 163, 1687–1696. doi: 10.1016/j.ijbiomac.2020.09.138.

Vivek-Ananth, R. P., Krishnaswamy, S., and Samal, A. (2021). Potential phytochemical inhibitors of SARS-CoV-2 helicase Nsp13: a molecular docking and dynamic simulation study. *Mol. Divers*. doi: 10.1007/s11030-021-10251-1.

White, M. A., Lin, W., and Cheng, X. (2020). Discovery of COVID-19 Inhibitors Targeting the SARS-CoV-2 Nsp13 Helicase. *J. Phys. Chem. Lett.* 11, 9144–9151. doi: 10.1021/acs.jpclett.0c02421.

Yang, N., Tanner, J. A., Wang, Z., Huang, J.-D., Zheng, B.-J., Zhu, N., et al. (2007a). Inhibition of SARS coronavirus helicase by bismuth complexes. *Chem. Commun.* 4413–4415. doi: 10.1039/B709515E.

Yang, N., Tanner, J. A., Zheng, B.-J., Watt, R. M., He, M.-L., Lu, L.-Y., et al. (2007b). Bismuth Complexes Inhibit the SARS Coronavirus. *Ang. Chem. Int. Ed.* 46, 6464–6468. doi: 10.1002/anie.200701021.

Yu, M.-S., Lee, J., Lee, J. M., Kim, Y., Chin, Y.-W., Jee, J.-G., et al. (2012). Identification of myricetin and scutellarein as novel chemical inhibitors of the SARS coronavirus helicase, nsP13. *Bioorg. Med. Chem. Lett*. 22, 4049–4054. doi: 10.1016/j.bmcl.2012.04.081.

Yuan, S., Wang, R., Chan, J. F.-W., Zhang, A. J., Cheng, T., Chik, K. K.-H., et al. (2020). Metallodrug ranitidine bismuth citrate suppresses SARS-CoV-2 replication and relieves virus-associated pneumonia in Syrian hamsters. *Nat. Microbiol.* 5, 1439–1448. doi: 10.1038/s41564-020-00802-x.

Zeng, J., Weissmann, F., Bertolin, A. P., Posse, V., Canal, B., Ulferts, R., et al. (2021). Identifying SARS-CoV-2 antiviral compounds by screening for small molecule inhibitors of nsp13 helicase. *Biochem. J.* 478, 2405–2423. doi: 10.1042/BCJ20210201.

Zhang, S., Huang, W., Ren, L., Ju, X., Gong, M., Rao, J., et al. (2022). Comparison of viral RNA–host protein interactomes across pathogenic RNA viruses informs rapid antiviral drug discovery for SARS-CoV-2. *Cell. Res.* 32, 9–23. doi: 10.1038/s41422-021-00581-y.

Zhao, S., Kanno, Y., Li, W., Wakatabi, H., Sasaki, T., Koike, K., et al. (2016). Picrasidine N Is a Subtype-Selective PPARβ/δ Agonist. *J. Nat. Prod.* 79, 879–885. doi: 10.1021/acs.jnatprod.5b00909.

Zia, M., Muhammad, S., Shafiq-urRehman, Bibi, S., Abbasi, S. W., Al-Sehemi, A. G., et al. (2021). Exploring the potential of novel phenolic compounds as potential therapeutic candidates against SARS-CoV-2, using quantum chemistry, molecular docking and dynamic studies. *Bioorg. Med. Chem. Lett.* 43, 128079. doi: 10.1016/j.bmcl.2021.128079.
